# Prevalence of Signs of Severity Identified in the Thai Population with Malaria: A Systematic Review and Meta-Analysis

**DOI:** 10.3390/ijerph19031196

**Published:** 2022-01-21

**Authors:** Wanida Mala, Polrat Wilairatana, Chutharat Samerjai, Frederick Ramirez Masangkay, Kwuntida Uthaisar Kotepui, Manas Kotepui

**Affiliations:** 1 Medical Technology, School of Allied Health Sciences, Walailak University, Tha Sala, Nakhon Si Thammarat 80160, Thailand; wanida.ma@wu.ac.th (W.M.); chutharat.sm@wu.ac.th (C.S.); kwuntida.ut@wu.ac.th (K.U.K.); 2 Department of Clinical Tropical Medicine, Faculty of Tropical Medicine, Mahidol University, Bangkok 10400, Thailand; polrat.wil@mahidol.ac.th; 3 Department of Medical Technology, Faculty of Pharmacy, University of Santo Tomas, Manila 1008, Philippines; frederick_masangkay2002@yahoo.com

**Keywords:** malaria, *Plasmodium falciparum*, Thailand, signs of severity, severe malaria, complicated malaria

## Abstract

Understanding the prevalence of signs of severity identified in the Thai population with malaria could aid clinical management and disease control efforts, decrease mortality, and promote malaria elimination in Thailand. This systematic review aimed to collate the evidence regarding signs of severity identified in the Thai population with malaria. MEDLINE, Web of Science, and Scopus were searched for potentially relevant studies. The quality of the included studies was assessed using the Joanna Briggs Institute critical appraisal tools. The pooled prevalence of signs of severity among patients with severe malaria and the pooled proportion of each sign of severity among all signs of severity were estimated using random-effects models. Heterogeneity among included studies was assessed using Cochran’s Q test. A subgroup analysis was performed to evaluate whether differences in pooled estimates between different study sites. Publication bias was assessed by visualizing funnel plot asymmetry and using Egger’s test. Among 741 studies identified by literature searching, 12 studies of a total of 2900 patients with severe malaria, in 7 Thai hospitals, met the eligibility criteria. Results of meta-analyses showed that the signs of the severity of malaria with the highest prevalence in Thailand were jaundice (54%), hyperparasitemia (47%), impaired consciousness/coma (21%), acidosis (18%), renal impairment (13%), shock (10%), convulsions (9%), severe anemia (8%), pulmonary edema/acute respiratory distress syndrome (ARDS) (8%), hypoglycemia (4%), and bleeding/disseminated intravascular coagulation (DIC) (2%). The signs of the severity of malaria that made up the highest proportion of all signs of severity identified in the Thai population with malaria were hyperparasitemia (33%), jaundice (33%), impaired consciousness/coma (12%), acidosis (9%), renal impairment (7%), severe anemia (6%), convulsions (5%), shock (5%), pulmonary edema/ARDS (3%), bleeding/DIC (1%), and hypoglycemia (1%). The present study revealed the prevalence of signs of severity identified in the Thai population with malaria. Jaundice, hyperparasitemia, and impaired consciousness/coma were the most common signs of severity identified. These results may inform the management of patients with severe malaria and promote malaria-elimination efforts in Thailand.

## 1. Background

Malaria is a life-threatening disease caused by members of the protozoan genus *Plasmodium* that are transmitted to humans via the bites of infected female *Anopheles* mosquitoes [1]. Globally, approximately 229,000 cases of malaria and 409,000 deaths were reported in 2019 [1]. Five *Plasmodium* species (*P*. *falciparum*, *P*. *vivax*, *P*. *malariae*, *P*. *ovale*, and *P*. *knowlesi*) most commonly infect humans [2,3]. In some countries in South East Asia where humans inhabit forested regions, *P*. *cynomolgi*—a parasite that primarily infects non-human primates—is being detected more frequently in humans [4]. Infection of *Plasmodium* parasites can lead to different clinical outcomes depending on pre-existing partial immunity to malaria. In moderate- to high-transmission areas, clinical disease is confined mainly to young children who have no immunity to malaria, whereas adolescents and adults with partial immunity have less severe clinical outcomes. In contrast, in low-transmission areas, both children and adults alike suffer from malaria symptoms and are at risk of severe malaria if left untreated [5].

According to the recent World Health Organization (WHO) guidelines for malaria, severe malaria is defined as the presence of *P*. *falciparum* asexual parasitemia with one or more of the following signs of severity: impaired consciousness, prostration, multiple convulsions, acidosis, hypoglycemia, severe malarial anemia, renal impairment, jaundice, pulmonary edema, significant bleeding, shock/hypotension, and hyperparasitemia (*P*. *falciparum* parasitemia > 10%) [5]. Although severe malaria is mainly caused by *P*. *falciparum*, fewer patients develop severe malaria following *P*. *knowlesi*, *P*. *vivax*, *P*. *malariae*, and *P*. *ovale* infection [3,6,7,8]. Severe malaria left untreated can lead to a high rate of mortality, while prompt and effective treatment can decrease the risk of mortality to 10–20% [5]. Nevertheless, mortality risk may depend on the types of signs of severity: for instance, higher mortality rates may be associated with acidosis, whereas lower mortality rates may be associated with severe anemia [5]. Moreover, mortality risk depends on the infecting *Plasmodium* species, the degree of vital organ dysfunction, age, background immunity, co-morbidities, and co-infections [9,10,11].

Thailand is one of the six countries of the Greater Mekong subregion and is bordered by Myanmar, Laos, Cambodia, and Malaysia. Malaria cases and deaths in Thailand have continuously decreased from 81,692 cases and 625 deaths in 2000 to 3538 cases and 13 deaths in 2019 [1]. Approximately 32 million Thai individuals are at risk of malaria, particularly near the Thai–Cambodia border, an area with high transmission of multidrug-resistant *P*. *falciparum* malaria [12]. Based on the basis of the recent weekly reports from the Department of Disease Control, Ministry of Public Health, Thailand, the most serious burdens of malaria were reported in three provinces along the Thai–Burma border: Tak (1087 cases), Yala (905 cases), and Kanchanaburi (468 cases) [13]. In Thailand, malaria transmission was low overall, but individuals of all ages may develop severe malaria [14]. A previous study conducted near the Western border of Thailand showed that the risks of developing severe malaria and death steadily declined with age [14]. Another study conducted in the same area showed that male sex, younger age, Karen ethnicity, and forest-related occupations were risk factors for malaria [15]. A recent study conducted in southern Thailand showed that detecting *Plasmodium* parasites in blood samples (prevalence 0.45–0.61%) was associated with male sex and staying outdoors at night [16].

A better understanding of the signs of severity identified in the Thai population with malaria could inform the clinical management of patients with severe malaria and decrease mortality. To the best of our knowledge, a systematic review of the characteristics of signs of severity of malaria has not been conducted in Thailand. Therefore, the present systematic review aimed to collate the evidence regarding signs of severity identified in the Thai population with malaria. The results of this study will help to better understand the signs of severity in the Thai population with malaria and will serve as a guide for clinicians to recognize and treat these signs of severity. These data may also contribute toward better management and control of malaria, with the eventual goal of malaria elimination in Thailand.

## 2. Methods

### 2.1. Protocol and Registration

The reporting of this systematic review and meta-analysis followed the Preferred Reporting Items for Systematic Reviews and Meta-Analyses (PRISMA) [17]. The protocol of systematic review was registered at PROSPERO (ID: CRD42021283237).

### 2.2. Search Strategy

MEDLINE, Web of Science, and Scopus were searched for relevant studies using a combination of search terms “(“severe malaria” OR “complicated malaria”) AND (Thai OR Thailand OR Siam)” from inception to 8 August 2021 (Table S1). Additional searching for relevant studies using Google Scholar was performed to ensure that all potentially relevant studies were included in the search results.

### 2.3. Eligibility Criteria

Eligibility criteria were based on a PICo (participants, outcome of interest, context) framework. Participants were patients with severe *P*. *falciparum* malaria in all age groups according to WHO guidelines [5]. The outcome of interest was the prevalence of signs of severity identified in the Thai population with malaria. The context was Thailand. Therefore, the inclusion criteria for studies were as follows: (1) studies enrolled patients with severe *P*. *falciparum* malaria; (2) studies must be conducted in Thailand; (3) any study design was considered; and (4) studies reported signs of severity among patients with malaria. The exclusion criteria were as follows: (1) duplicate studies using the same groups of participants; (2) case reports of severe malaria; (3) studies of uncomplicated malaria; (4) studies that do not provide data on signs of severity; (5) short reports, (6) studies with no full-text available; and (7) review articles.

### 2.4. Study Selection

Potentially relevant studies were selected according to the eligibility criteria. After duplicate studies were removed, the titles and abstracts of the remaining studies were screened, and irrelevant studies were excluded. Next, the full texts of the remaining studies were examined for eligibility, and studies that did not meet the eligibility criteria were excluded. The reason for exclusion was noted. The studies that met the eligibility criteria were included for further analysis. Study selection was performed by two authors independently. Disagreements between authors in study selection were resolved by discussion.

### 2.5. Data Extraction

The following data were extracted from each study into a standardized spreadsheet: name of the first author, publication year, year the study was conducted, study sites, study design, number of participants, age groups included, percentage of male participants, number of patients who died, number of patients with each sign of severity, and the total number of signs of severity. Data were extracted by two authors. Disagreements between authors in data extraction were resolved by discussion.

### 2.6. Quality of the Included Studies

The quality of the included studies was assessed using the Joanna Briggs Institute (JBI) critical appraisal tools [18]. For clinical trials, the JBI critical appraisal checklist for randomized controlled trials (13 criteria) was used. For observational studies, the JBI critical appraisal checklist for studies reporting prevalence data (nine criteria) was used. A score of more than 75% of the total indicated a high-quality study, whereas a score less than 75% of the total indicated a moderate- or low-quality study. Moderate- or low-quality studies were retained for qualitative synthesis.

### 2.7. Data Synthesis

The pooled prevalence of signs of severity identified in the Thai population with malaria and the pooled proportion of each sign of severity among all signs of severity were estimated using random-effects models assuming that the outcomes of the included studies were heterogeneous. Heterogeneity among the included studies was assessed using Cochran’s Q test. Values of *p* < 0.01 or I^2^ more than 50% indicated substantial heterogeneity among the included studies. If the outcome of the included studies was homogenous (no substantial heterogeneity), data were pooled using fixed-effects models. A subgroup analysis was performed to evaluate differences in pooled estimates between different study sites. Publication bias was assessed by visualizing funnel plot asymmetry and using Egger’s test. If the funnel plot was asymmetrical or Egger’s test returned a value of *p* < 0.05, contour-enhanced funnel plots were evaluated to identify potential sources of funnel plot asymmetry. All analyses were performed using Stata version 14.2 (StataCorp, College Station, TX, USA).

## 3. Results

### 3.1. Search Results

**Figure 1:**
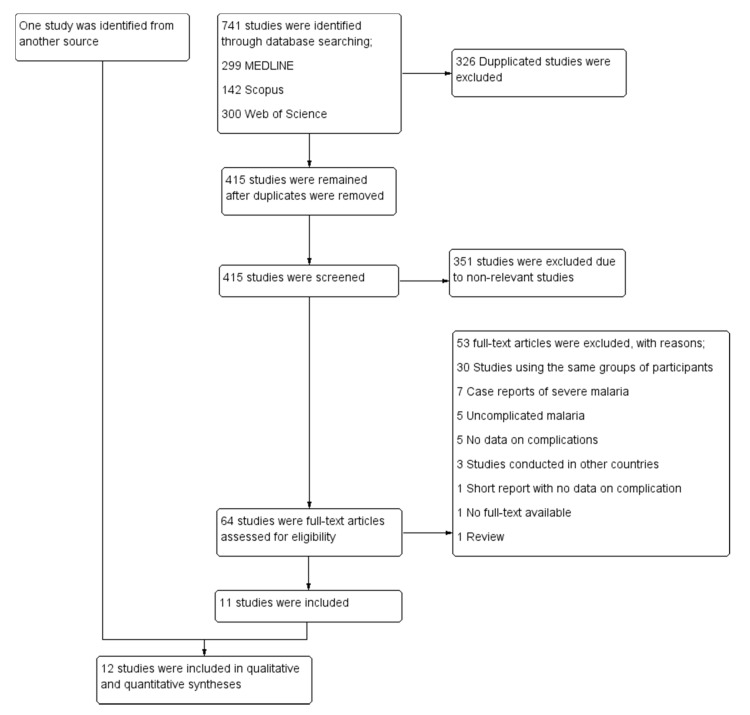
Study selection.

A total of 741 studies were identified from the 3 databases: 299 from MEDLINE, 142 from Scopus, and 300 from Web of Science. After 326 duplicates were removed, the titles and abstracts of the 415 remaining studies were screened, and 351 irrelevant studies were excluded. The full texts of 64 studies were examined, and 53 studies were excluded for the following reasons: 30 studies used the same group of participants, 7 studies were case reports of severe malaria, 5 studies were of uncomplicated malaria, 5 studies did not report data on signs of severity, 3 studies were conducted in other countries, 1 short report did not provide data on signs of severity, 1 study had no full-text available, and 1 study was a review article. Eleven studies [14,19,20,21,22,23,24,25,26,27,28] met the eligibility criteria and were included in the meta-analysis. One additional study [29] was identified through another source (Google Scholar) and was included in the meta-analysis. Finally, 12 studies [14,19,20,21,22,23,24,25,26,27,28,29] were included in the qualitative and quantitative syntheses (Figure 1).

### 3.2. Characteristics of the Included Studies

**Figure 2:**
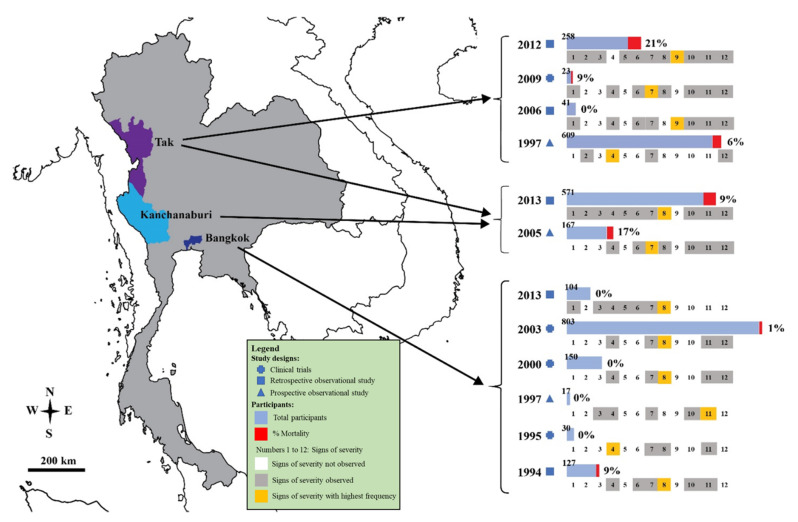
Details of signs of severity identified in the Thai population with malaria. Signs of severity: 1 acidosis; 2 convulsions; 3 DIC/bleeding; 4 hyperparasitemia; 5 hypoglycemia; 6 hypotension/shock; 7 impaired consciousness/coma; 8 jaundice; 9 prostrations; 10 pulmonary edema/ARDS; 11 renal impairments; 12 severe anemia. The years indicated are the years of publication.

The characteristics of the included studies are shown in Table 1. The included studies were published between 1994 and 2013 and were conducted between 1991 and 2012. Most of the included studies were conducted at the Hospital for Tropical Diseases (Bangkok province) (5/12, 41.7%), whereas other studies were conducted at Mae Sot Hospital (Tak province) [19,28], Shoklo Hospital (Tak province) [14], Somdejt Prachaotaksin Maharaj Hospital (Tak province) [25], Sangklaburi Hospital (Kanchanaburi province), Mae Sot Hospital (Tak province) [21], Ramathibodi Hospital (Bangkok province), Pramongkutklao Hospital (Bangkok province), and hospitals in Kanchanaburi (Kanchanaburi province), Sangklaburi (Kanchanaburi province), and Mae Sot (Tak province) [24]. The designs of the included studies included retrospective observational studies [24,25,28,29], clinical trials [19,22,23,27], and prospective observational studies [14,20,21]. Most of the included studies [19,20,21,22,23,24,26,28] examined severe *P*. *falciparum* malaria in adult patients (8/12, 66.7%), whereas the remaining studies examined severe malaria in both children and adults [14,27,29] or only children [25]. Included studies were conducted in three provinces (Figure 2). A total of 2900 patients with severe *P*. *falciparum* malaria were included in the meta-analysis.

### 3.3. Methodological Quality of the Included Studies

For clinical trials, one study was a high-quality study, while three studies were moderate- or low-quality studies [22,23,27]. For observational studies, all eight studies had scores of more than 75% of the total indicated high-quality studies [14,19,20,21,24,25,26,28,29]. Moderate- or low-quality studies were retained in the present systematic review for quantitative and qualitative syntheses.

### 3.4. Jaundice

**Figure 3:**
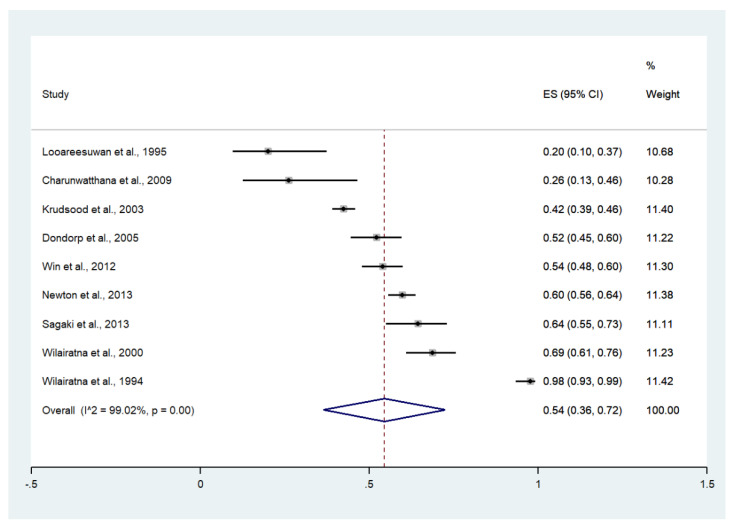
The pooled prevalence of jaundice among patients with severe malaria in Thailand. The label for the x-axis is the scale of estimated prevalence. Abbreviations; ES, estimated prevalence; CI, confidence interval.

The meta-analysis of the results of nine studies that reported jaundice as a sign of severity, including a total of 2233 patients, showed that the pooled prevalence of jaundice among patients with severe malaria in Thailand was 54% (9 studies, 1213/2233 cases; 95% confidence interval (CI): 36–72%, I^2^: 99.02%). The lowest prevalence of jaundice (20%, 95% CI: 10–37%) was reported in a study conducted at the Hospital for Tropical Diseases in 1994 [23]. The highest prevalence of jaundice (98%, 95% CI: 93–99%) was reported in a study conducted at the Hospital for Tropical Diseases in 1991 [29] (Figure 3).

**Figure 4:**
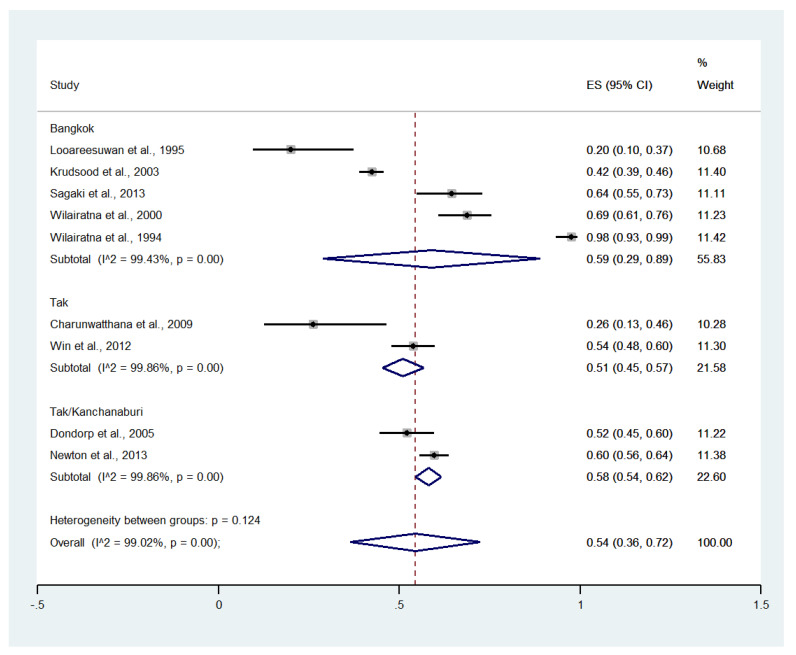
Subgroup analysis of the pooled prevalence of jaundice among patients with severe malaria in Thailand. The label for the x-axis is the scale of estimated prevalence. Abbreviations; ES, estimated prevalence; CI, confidence interval.

Subgroup analysis by province showed that the highest pooled prevalence of jaundice among patients with severe malaria was observed in Bangkok (59%, five studies; 95% CI: 29–89%, I^2^: 99.43%). A lower pooled prevalence of jaundice was observed in Tak/Kanchanaburi (58%, two studies; 95% CI: 54–62%, I^2^: 99.86%) and Tak (51%, two studies; 95% CI: 45–57%, I^2^: 99.86%) (Figure 4).

Meta-analysis of the results of nine studies, including a total of 3552 signs of severity, showed that the pooled proportion of jaundice among all signs of severity was 33% (nine studies, 1213/3552 signs of severity; 95% CI: 24–43%, I^2^: 97.07%). The lowest proportion of jaundice (16%, 95% CI: 8–31%) was reported in a study conducted at Mae Sot Hospital from 2003 to 2005 [19]. The highest proportion of jaundice (54%, 95% CI: 47–61%) was reported in a study conducted at the Hospital for Tropical Diseases in 1999 [21] (Supplementary Figure S1).

Subgroup analysis by province showed that the highest pooled proportion of jaundice was observed in Bangkok (40%, five studies; 95% CI: 30–50%, I^2^: 92.26%). Lower pooled proportions of jaundice were observed in Tak/Kanchanaburi (37%, two studies; 95% CI: 34–40%, I^2^: 97.16%) and Tak (17%, two studies; 95% CI: 14–19%, I^2^: 99.56%) (Supplementary Figure S2).

### 3.5. Hyperparasitemia

**Figure 5:**
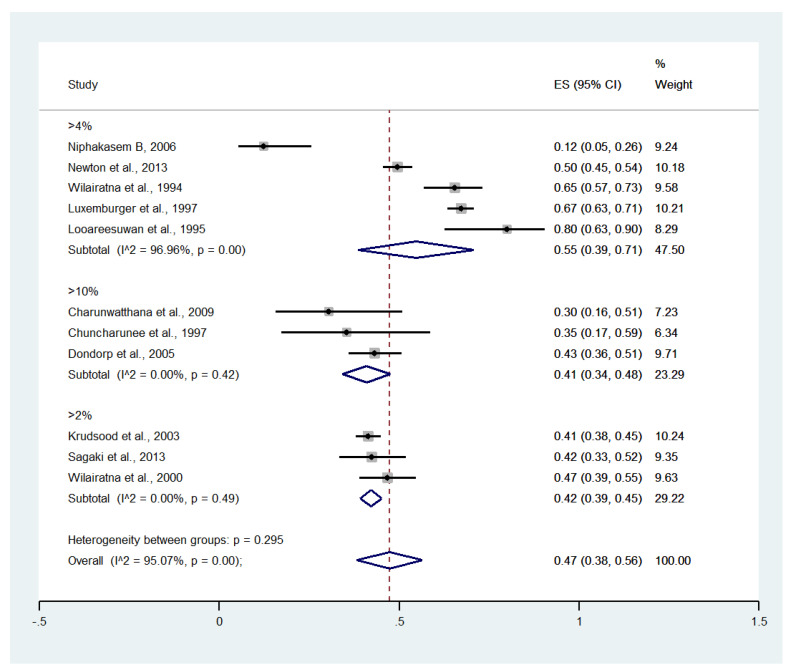
The pooled prevalence of hyperparasitemia among patients with severe malaria in Thailand at different cutoffs. The label for the x-axis is the scale of estimated prevalence. Abbreviations; ES, estimated prevalence; CI, confidence interval.

Meta-analysis of the results of 11 studies, including a total of 2642 patients, showed that the pooled prevalence of hyperparasitemia among patients with severe malaria in Thailand was 47% (11 studies, 1335/2642 cases; 95% CI: 38–56%, I^2^: 95.07%). Subgroup analyses of hyperparasitemia among patients with severe malaria defined using cutoffs of >2%, >4%, and >10% showed a pooled prevalence of 42% (three studies, 446/1057 cases; 95% CI: 39–45%, I^2^: 0%), 55% (five studies, 804/1378 cases; 95% CI: 39–71%, I^2^: 96.96%), and 41% (three studies, 85/207 cases; 95% CI: 34–48%, I^2^: 0%), respectively (Figure 5).

Meta-analysis of the results of 11 studies, including a total of 3498 signs of severity, showed that the pooled proportion of hyperparasitemia among all signs of severity was 33% (11 studies, 1335/3498 signs of severity; 95% CI: 25–42%, I^2^: 96.47%). Subgroup analysis of the proportion of hyperparasitemia using different cutoffs (>2%, >4%, >10%) was performed. The pooled proportion of hyperparasitemia among all signs of severity using cutoffs of >2%, >4%, and >10% was 32% (three studies, 446/1281 among all signs of severity; 95% CI: 20–44%, I^2^: 94.20%), 42% (five studies, 804/1843 among all signs of severity; 95% CI: 26–58%, I^2^: 97.72%), and 23% (three studies, 85/374 among all signs of severity; 95% CI: 18–27%, I^2^: 0%), respectively (Supplementary Figure S3).

### 3.6. Impaired Consciousness

**Figure 6:**
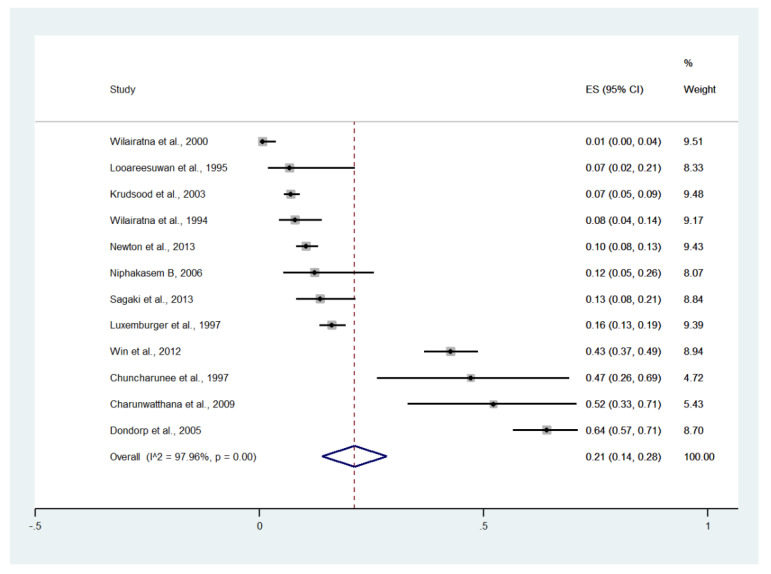
The pooled prevalence of impaired consciousness among patients with severe malaria in Thailand. The label for the x-axis is the scale of estimated prevalence. Abbreviations; ES, estimated prevalence; CI, confidence interval.

Meta-analysis of the results of 12 studies, including a total of 2900 patients, showed that the pooled prevalence of impaired consciousness among patients with severe malaria in Thailand was 21% (12 studies, 1213/2900 cases; 95% CI: 14–28%, I^2^: 97.96%). The lowest prevalence of impaired consciousness was reported in a study conducted at the Hospital for Tropical Diseases in 1999 (1%, 95% CI: 0–4%) [27]. The highest prevalence of impaired consciousness was reported in a study conducted at Sangklaburi Hospital, Mae Sot Hospital (21%, 95% CI: 14–28%) [29] (Figure 6).

**Figure 7:**
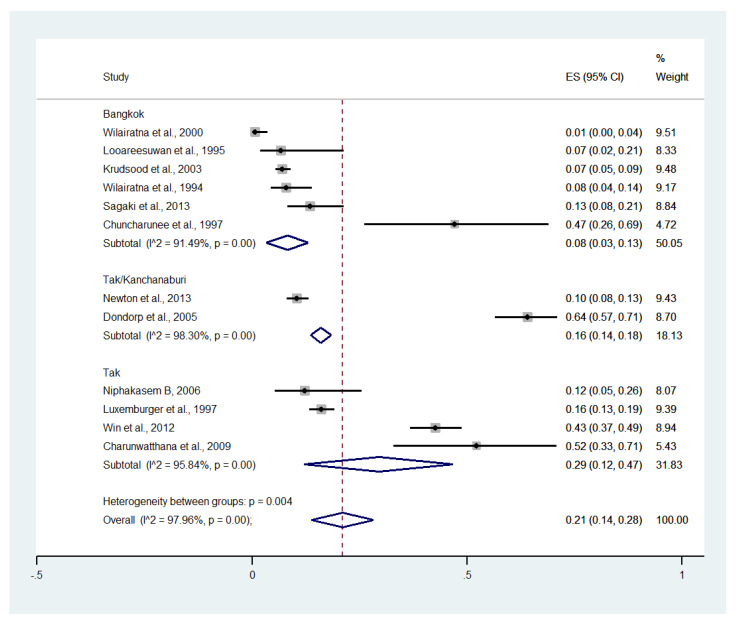
Subgroup analysis of the pooled prevalence of impaired consciousness among patients with severe malaria in Thailand. The label for the x-axis is the scale of estimated prevalence. Abbreviations; ES, estimated prevalence; CI, confidence interval.

Subgroup analysis by province showed that the highest pooled prevalence of im-paired consciousness among patients with severe malaria was observed in Tak (29%, five studies; 95% CI: 12–47%, I^2^: 95.84%). A lower pooled prevalence of impaired consciousness was observed in Tak/Kanchanaburi (16%, two studies; 95% CI: 14–18%, I^2^: 98.30%) and Bangkok (8%, six studies; 95% CI: 3–13%, I^2^: 91.49%) (Figure 7).

Meta-analysis of the results of 12 studies, including a total of 4324 among all signs of severity, showed that the pooled proportion of impaired consciousness among all signs of severity was 12% (12 studies, 482/4324 signs of severity; 95% CI: 8–16%, I^2^: 96.55%). The lowest proportion of impaired consciousness was reported in a study conducted at the Hospital for Tropical Diseases in 1999 (1%, 95% CI: 0–3%) [27]. The highest proportion of impaired consciousness was reported in a study conducted at Sangklaburi Hospital and Mae Sot Hospital (35%, 95% CI: 30–40%) [21] (Supplementary Figure S4).

Subgroup analysis by province showed that the highest pooled proportion of impaired consciousness was observed in Tak (14%, four studies; 95% CI: 11–17%, I^2^: 54.42%). The lower pooled proportions of impaired consciousness were observed in Tak/Kanchanaburi (10%, six studies; 95% CI: 8–11%, I^2^: 98.24%) and Bangkok (5%, six studies; 95% CI: 2–9%, I^2^: 90.55%) (Supplementary Figure S5).

### 3.7. Acidosis

**Figure 8:**
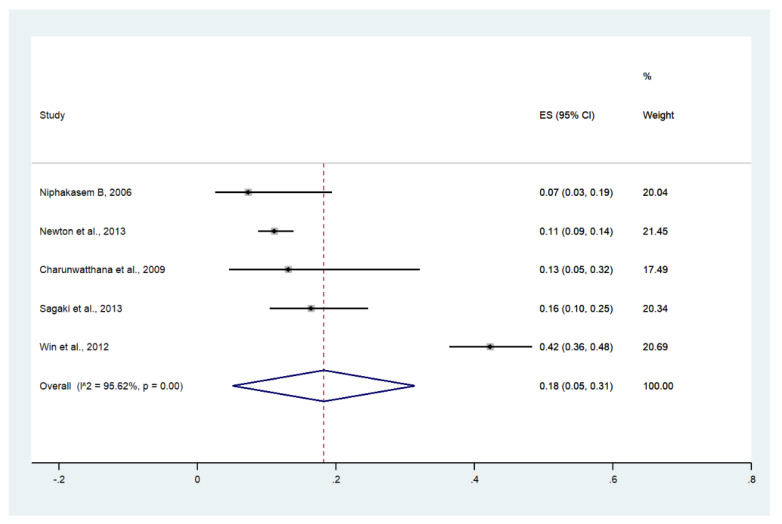
The pooled prevalence of acidosis among patients with severe malaria in Thailand. The label for the x-axis is the scale of estimated prevalence. Abbreviations; ES, estimated prevalence; CI, confidence interval.

Meta-analysis of the results of five studies, including a total of 997 patients, showed that the pooled prevalence of acidosis among patients with severe malaria in Thailand was 18% (five studies, 195/997 cases; 95% CI: 5–31%, I^2^: 95.62%). The lowest prevalence of acidosis was reported in a study conducted at Somdejt Prachaotaksin Maharaj Hospital from 2003 to 2006 (7%, 95% CI: 3–19%) [25]. The highest prevalence of acidosis was reported in a study conducted at Mae Sot Hospital from 2004 to 2008 (18%, 95% CI: 5–31%) [28] (Figure 8).

Meta-analysis of the results of five studies, including a total of 1966 among all signs of severity, showed that the pooled proportion of acidosis among all signs of severity was 9% (five studies, 195/1966 signs of severity; 95% CI: 6–12%, I^2^: 75.27%). The lowest proportion of acidosis was reported in a study conducted at Somdejt Prachaotaksin Maharaj Hospital from 2003 to 2006 (16%, 95% CI: 8–31%) [25]. The highest proportion of impaired consciousness was reported in a study conducted at Mae Sot Hospital from 2004 to 2008 (54%, 95% CI: 47–61%) [28] (Supplementary Figure S6).

### 3.8. Renal Impairment

**Figure 9:**
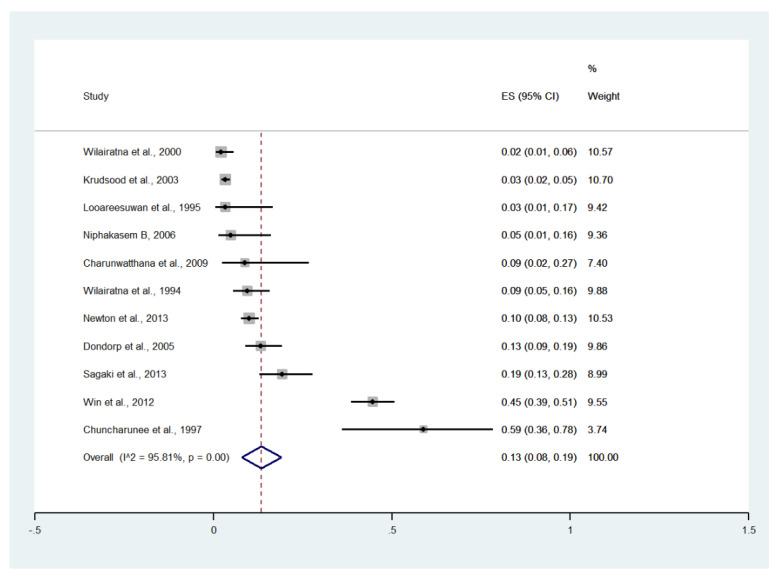
The pooled prevalence of renal impairment among patients with severe malaria in Thailand. The label for the x-axis is the scale of estimated prevalence. Abbreviations; ES, estimated prevalence; CI, confidence interval.

Meta-analysis of the results of 11 studies, including a total of 2291 patients, showed that the pooled prevalence of renal impairment among patients with severe malaria in Thailand was 13% (11 studies, 270/2291 cases; 95% CI: 8–19%, I^2^: 95.81%). The lowest prevalence of renal impairment was reported in a study conducted at the Hospital for Tropical Diseases in 1999 (2%, 95% CI: 1–6%) [27]. The highest prevalence of renal impairment was reported in a study conducted at Ramathibodi Hospital and Pramongkutklao Hospital from 1992 to 1994 (59%, 95% CI: 36–78%) [20] (Figure 9).

**Figure 10:**
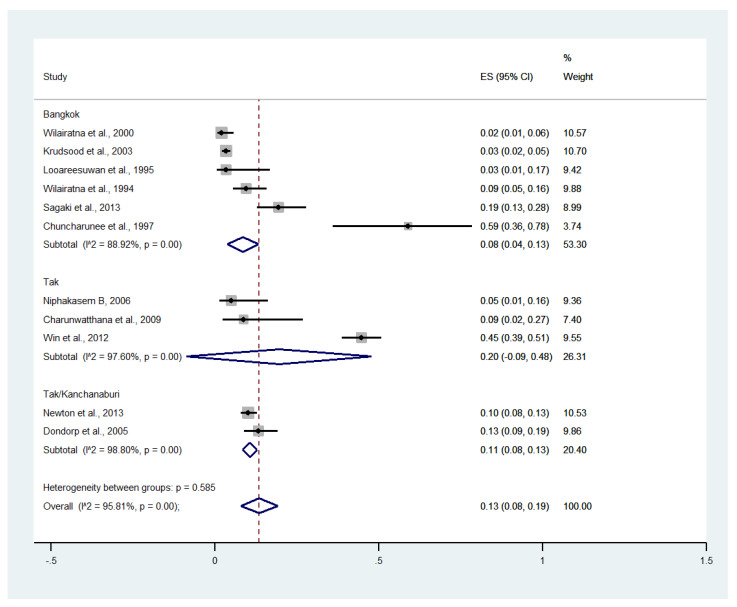
Subgroup analysis of the pooled prevalence of renal impairment among patients with severe malaria in Thailand. The label for the x-axis is the scale of estimated prevalence. Abbreviations; ES, estimated prevalence; CI, confidence interval.

Subgroup analysis by province showed that the highest pooled prevalence of renal impairment among patients with severe malaria was observed in Tak (20%, five studies; 95% CI: −9–48%, I^2^: 97.60%). The lower pooled prevalence of renal impairment was observed in Tak/Kanchanaburi (11%, two studies; 95% CI: 8–13%, I^2^: 98.8%) and Bangkok (8%, six studies; 95% CI: 4–13%, I^2^: 88.92%) (Figure 10).

Meta-analysis of the results of 11 studies, including a total of 3630 signs of severity, showed that the pooled proportion of renal impairment among all signs of severity was 7% (11 studies, 270/3630 signs of severity; 95% CI: 4–9%, I^2^: 90.32%). The lowest proportion of renal impairment was reported in a study conducted at the Hospital for Tropical Diseases in 1999 (2%, 95% CI: 1–5%) [27]. The highest proportion of renal impairment was reported in a study conducted at Ramathibodi Hospital and Pramongkutklao Hospital from 1992 to 1994 (32%, 95% CI: 19–50%) [21] (Supplementary Figure S7).

Subgroup analysis by province showed that the highest pooled proportion of renal impairment was observed in Tak (8%, three studies; 95% CI: 1–16%, I^2^: 84.14%). Lower pooled proportions of renal impairment were observed in Tak/Kanchanaburi (7%, three studies; 95% CI: 5–8%, I^2^: 92.07%) and Bangkok (5%, six studies; 95% CI: 2–8%, I^2^: 80.98%) (Supplementary Figure S8).

### 3.9. Severe Anemia

**Figure 11:**
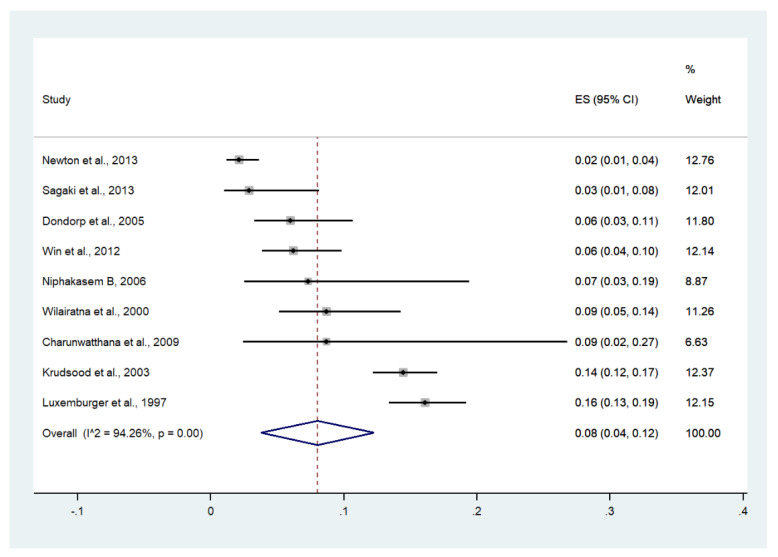
The pooled prevalence of severe anemia among patients with severe malaria in Thailand. The label for the x-axis is the scale of estimated prevalence. Abbreviations; ES, estimated prevalence; CI, confidence interval.

Meta-analysis of the results of nine studies, including a total of 2726 patients, showed that the pooled prevalence of severe anemia among patients with severe malaria in Thailand was 8% (nine studies, 273/2726 cases; 95% CI: 4–12%, I^2^: 94.26%). The lowest pooled prevalence of severe anemia was reported in a study conducted at hospitals in Kanchanaburi (1986 to 1993), hospitals in Sangklaburi (1994 to1995), and hospitals in Mae Sot (2%, 95% CI: 1–4%) [24]. The highest prevalence of severe anemia was reported in a study conducted at Shoklo Hospital in 1994 (16%, 95% CI: 13–19%) [14] (Figure 11).

**Figure 12:**
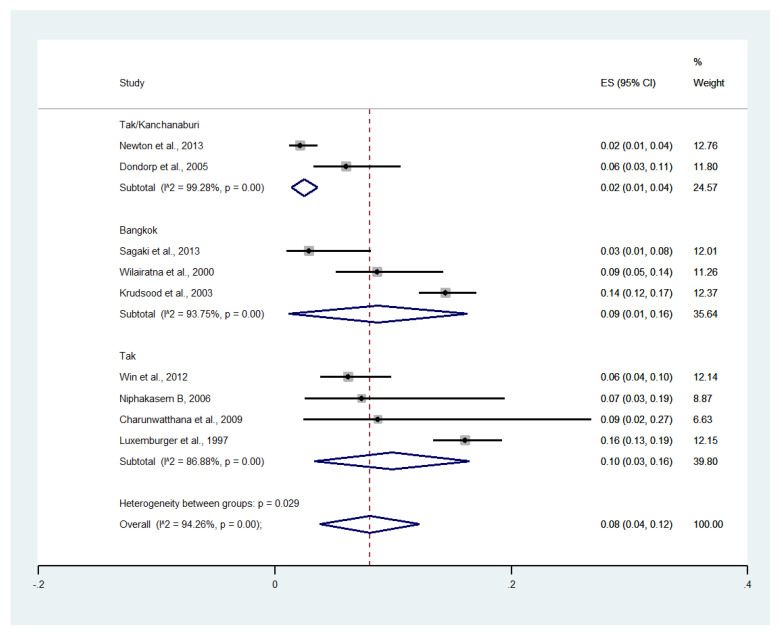
Subgroup analysis of the pooled prevalence of severe anemia among patients with severe malaria in Thailand. The label for the x-axis is the scale of estimated prevalence. Abbreviations; ES, estimated prevalence; CI, confidence interval.

Subgroup analysis by province showed that the highest pooled prevalence of severe anemia among patients with severe malaria was observed in Tak (10%, four studies; 95% CI: 3–16%, I^2^: 86.88%). The lower pooled prevalence of severe anemia was observed in Bangkok (9%, six studies; 95% CI: 1–16%, I^2^: 93.75%) and Tak/Kanchanaburi (2%, two studies; 95% CI: 1–4%, I^2^: 99.28%) (Figure 12).

Meta-analysis of the results of nine studies, including a total of 4026 signs of severity, showed that the pooled proportion of severe anemia among all signs of severity was 6% (nine studies, 273/4026 signs of severity; 95% CI: 3–9%, I^2^: 95.57%). The lowest proportion of severe anemia was reported in a study conducted at the Hospital for Tropical Diseases from 2006 to 2021 (1%, 95% CI: 0–4%) [26]. The highest proportion of severe anemia was reported in a study conducted at Shoklo Hospital in 1994 (14%, 95% CI: 12–17%) [14] (Supplementary Figure S9).

Subgroup analysis by province showed that the highest pooled proportion of severe anemia was observed in Tak (7%, four studies; 95% CI: 0–15%, I^2^: 96.06%). Lower pooled proportions of severe anemia were observed in Bangkok (7%, three studies; 95% CI: 0–15%, I^2^: 97.33%) and Tak/Kanchanaburi (2%, two studies; 95% CI: 1–2%, I^2^: 98.67%) (Supplementary Figure S10).

### 3.10. Convulsions

**Figure 13:**
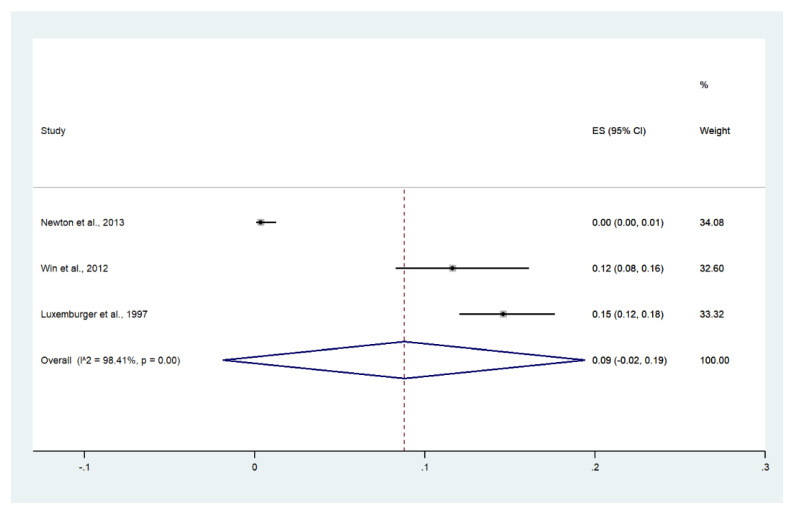
The pooled prevalence of convulsion among patients with severe malaria in Thailand. The label for the x-axis is the scale of estimated prevalence. Abbreviations; ES, estimated prevalence; CI, confidence interval.

Meta-analysis of the results of three studies, including a total of 1479 patients, showed that the pooled prevalence of convulsions among patients with severe malaria in Thailand was 9% (three studies, 121/1479 cases; 95% CI: 2–19%, I^2^: 98.41%). The lowest pooled prevalence of convulsions was reported in a study conducted at hospitals in Kanchanaburi (1986 to 1993), hospitals at Sangklaburi (1994 to 1995), and hospitals in Mae Sot (0%, 95% CI: 0–1%) [24]. The highest prevalence of convulsions was reported in a study conducted at Shoklo Hospital in 1994 (15%, 95% CI: 12–28%) [14] (Figure 13).

Meta-analysis of the results of three studies, including a total of 2355 signs of severity, showed that the pooled proportion of convulsion among all signs of severity was 5% (three studies, 121/2355 signs of severity; 95% CI: 0–11%, I^2^: 98.32%). The lowest proportion of convulsions was reported in a study conducted at hospitals in Kanchanaburi (1986 to 1993), hospitals at Sangklaburi (1994 to 1995), and hospitals in Mae Sot (1995 to 2002) (0%, 95% CI: 0–1%) [24]. The highest proportion of convulsions was reported in a study conducted at Shoklo Hospital in 1994 (5%, 95% CI: 0–11%) [14] (Supplementary Figure S11).

### 3.11. Shock

**Figure 14:**
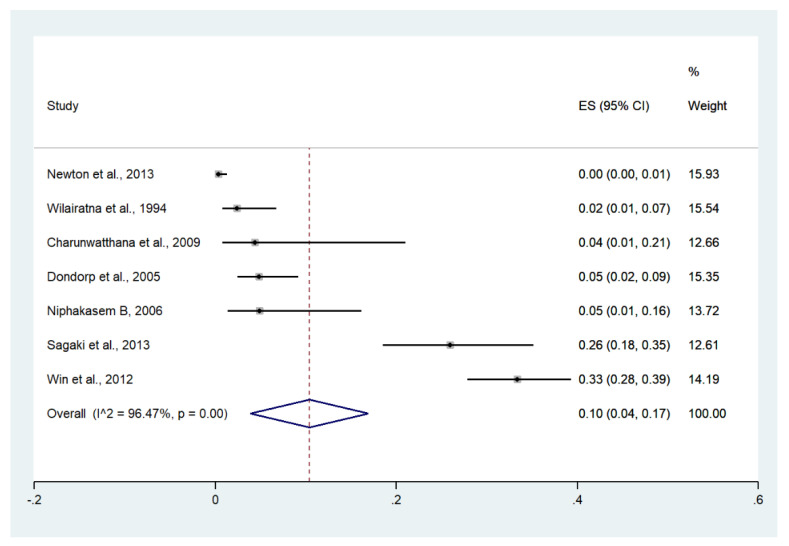
The pooled prevalence of shock among patients with severe malaria in Thailand. The label for the x-axis is the scale of estimated prevalence. Abbreviations; ES, estimated prevalence; CI, confidence interval.

Meta-analysis of the results of seven studies, including a total of 1291 patients, showed that the pooled prevalence of shock among patients with severe malaria in Thailand was 10% (seven studies, 129/1291 cases; 95% CI: 4–17%, I^2^: 96.47%). The lowest pooled prevalence of shock was reported in a study conducted at hospitals in Kanchanaburi (1986 to 1993), hospitals in Sangklaburi (1994 to 1995), and hospitals in Mae Sot (0%, 95% CI: 0–1%) [24]. The highest prevalence of shock was reported in a study conducted at Mae Sot Hospital from 2004 to 2008 (33%, 95% CI: 28–39%) [28] (Figure 14).

Meta-analysis of the results of seven studies, including a total of 2506 signs of se-verity, showed that the pooled proportion of shock among all signs of severity was 5% (seven studies, 129/2506 signs of severity; 95% CI: 2–8%, I2: 95.21%). The lowest proportion of shock was reported in a study conducted at hospitals in Kanchanaburi (1986 to 1993), hospitals in Sangklaburi (1994 to 1995), and hospitals in Mae Sot (1995 to 2002) (0%, 95% CI: 0–1%) [24]. The highest proportion of shock was reported in a study conducted at the Hospital for Tropical Diseases from 2006 to 2012 (12%, 95% CI: 9–17%) [26] (Supplementary Figure S12).

### 3.12. Pulmonary Edema/Acute Respiratory Distress Syndrome (ARDS)

**Figure 15:**
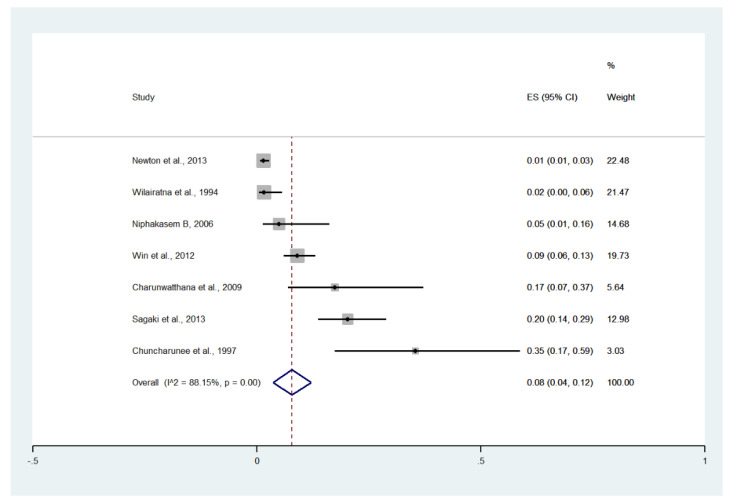
The pooled prevalence of pulmonary edema/ARDS among patients with severe malaria in Thailand. The label for the x-axis is the scale of estimated prevalence. Abbreviations; ES, estimated prevalence; CI, confidence interval.

Meta-analysis of the results of seven studies, including a total of 1141 patients, showed that the pooled prevalence of pulmonary edema/ARDS among patients with severe malaria in Thailand was 8% (seven studies, 66/1141 cases; 95% CI: 4–12%, I^2^: 88.15%). The lowest pooled prevalence of pulmonary edema/ARDS was reported in a study conducted at hospitals in Kanchanaburi (1986 to 1993), hospitals in Sangklaburi (1994 to 1995), and hospitals in Mae Sot (1%, 95% CI: 1–3%) [24]. The highest prevalence of pulmonary edema/ARDS was reported in a study conducted at Ramathibodi Hospital and Pramongkutklao Hospital from 1992 to 1994 (35%, 95% CI: 17–59%) [28] (Figure 15).

Meta-analysis of the results of seven studies, including a total of 1141 signs of severity, showed that the pooled proportion of pulmonary edema/ARDS among all signs of severity was 3% (seven studies, 66/1141 signs of severity; 95% CI: 1–5%, I^2^: 83.21%). The lowest proportion of pulmonary edema/ARDS was reported in a study conducted at the Hospital for Tropical Diseases in 1991 (1%, 95% CI: 0–3%) [29]. The highest proportion of pulmonary edema/ARDS was reported in a study conducted at Ramathibodi Hospital and Pramongkutklao Hospital from 1992 to 1994 (19%, 95% CI: 9–36%) [20] (Supplementary Figure S13).

### 3.13. Bleeding/Disseminated Intravascular Coagulation (DIC)

**Figure 16:**
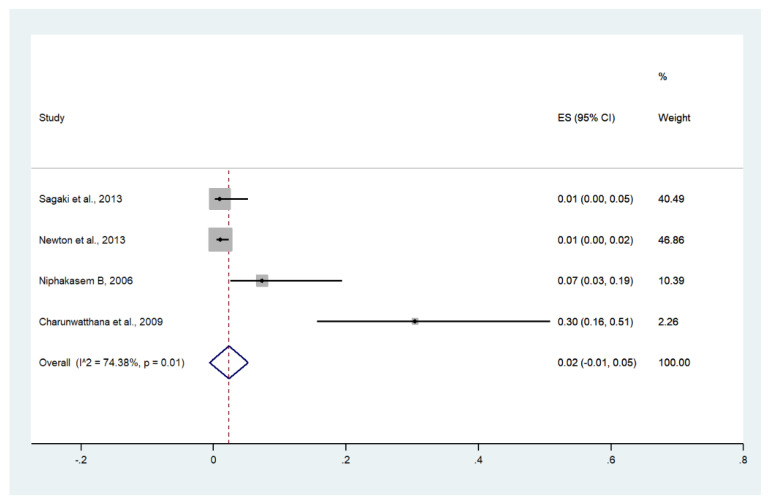
The pooled prevalence of bleeding/DIC among patients with severe malaria in Thailand. The label for the x-axis is the scale of estimated prevalence. Abbreviations; ES, estimated prevalence; CI, confidence interval.

Meta-analysis of the results of four studies, including a total of 950 patients with severe malaria, showed that the pooled prevalence of bleeding/DIC among patients with severe malaria in Thailand was 2% (four studies, 17/950 cases; 95% CI: −1–5%, I^2^: 74.38%). The lowest pooled prevalence of bleeding/DIC was reported in a study conducted at the Hospital for Tropical Diseases from 2006 to 2012 (1%, 95% CI: 0–5%) [26]. The highest prevalence of bleeding/DIC was reported in a study conducted at Ramathibodi Hospital and Pramongkutklao Hospital from 1992 to 1994 (30%, 95% CI: 16–51%) [28] (Figure 16).

Meta-analysis of the results of four studies, including a total of 1913 signs of severity, showed that the pooled proportion of bleeding/DIC among all signs of severity was 1% (four studies, 17/1913 signs of severity; 95% CI: 0–2%, I^2^: 52.7%). The lowest proportion of bleeding/DIC was reported in a study conducted at hospitals in Kanchanaburi (1986 to 1993), hospitals in Sangklaburi (1994 to 1995), and hospitals in Mae Sot (1995 to 2002) (0%, 95% CI: 0–1%) [24]. The highest proportion of bleeding/DIC was reported in a study conducted at Ramathibodi Hospital and Pramongkutklao Hospital from 1992 to 1994 (3%, 95% CI: 1–16%) [20] (Supplementary Figure S14).

### 3.14. Hypoglycemia

**Figure 17:**
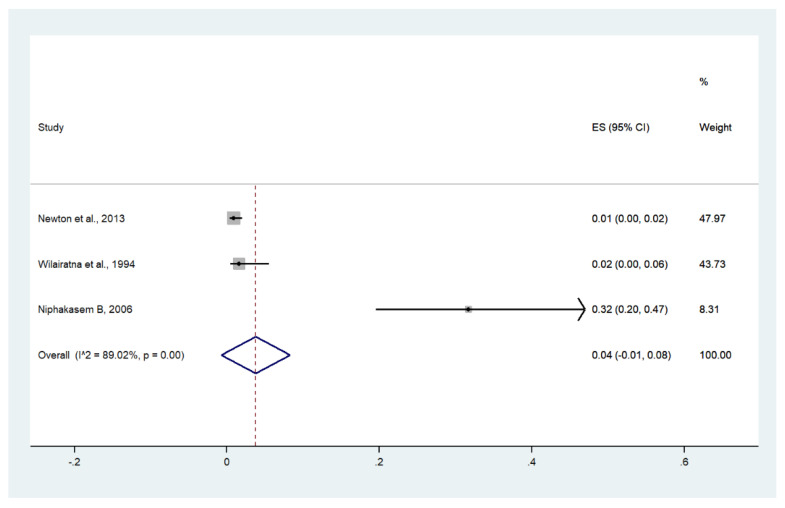
The pooled prevalence of hypoglycemia among patients with severe malaria in Thailand. The label for the x-axis is the scale of estimated prevalence. Abbreviations; ES, estimated prevalence; CI, confidence interval.

Meta-analysis of the results of three studies, including a total of 933 patients, showed that the pooled prevalence of hypoglycemia among patients with severe malaria in Thailand was 4% (three studies, 20/933 cases; 95% CI: −1–8%, I^2^: 89.02%). The lowest pooled prevalence of hypoglycemia was reported in a study conducted at hospitals in Kanchanaburi (1986 to 1993), hospitals in Sangklaburi (1994 to 1995), and hospitals in Mae Sot (1995 to 2002) (1%, 95% CI: 0–2%) [24]. The highest prevalence of hypoglycemia was reported in a study conducted at Somdejt Prachaotaksin Maharaj Hospital from 2003 to 2006 (32%, 95% CI: 20–47%) [28] (Figure 17).

Meta-analysis of the results of three studies, including a total of 1882 signs of severity, showed that the pooled proportion of hypoglycemia among all signs of severity was 1% (three studies, 20/1882 signs of severity; 95% CI: 0–2%, I2: 45.6%). The lowest proportion of hypoglycemia was reported in a study conducted at hospitals in Kanchanaburi (1986 to 1993), hospitals in Sangklaburi (1994 to 1995), and hospitals in Mae Sot (1995 to 2002) (1%, 95% CI: 0–1%) [24]. The highest proportion of hypoglycemia was reported in a study conducted at Mae Sot Hospital from 2004 to 2008 (3%, 95% CI: 1–16%) [20] (Supplementary Figure S15).

### 3.15. Prostration

Prostration was reported in two studies conducted at Somdejt Prachaotaksin Maharaj Hospital from 2003 to 2006 (24/41 cases, 58.5%) [25] and Mae Sot Hospital from 2004 to 2008 (178/258 cases, 69%) [28].

### 3.16. Prevalence and Proportion of Severe Malaria in Thailand

Of all patients with severe malaria, 54% of patients had jaundice, 47% had hyperparasitemia, 21% had impaired consciousness/coma, 18% had acidosis, 13% had renal impairment, 10% had shock, 9% had convulsions, 8% had severe anemia, 8% had pulmonary edema/ARDS, 4% had hypoglycemia, and 2% had bleeding/DIC (Table 2). Of all the reported signs of severity included in this meta-analysis, the most prevalent signs of severity were hyperparasitemia (33%), jaundice (33%), impaired consciousness/coma (12%), acidosis (9%), renal impairment (7%), severe anemia (6%), convulsions (5%), shock (5%), pulmonary edema/ARDS (3%), bleeding/DIC (1%), and hypoglycemia (1%) (Table 3).

### 3.17. Deaths

**Figure 18:**
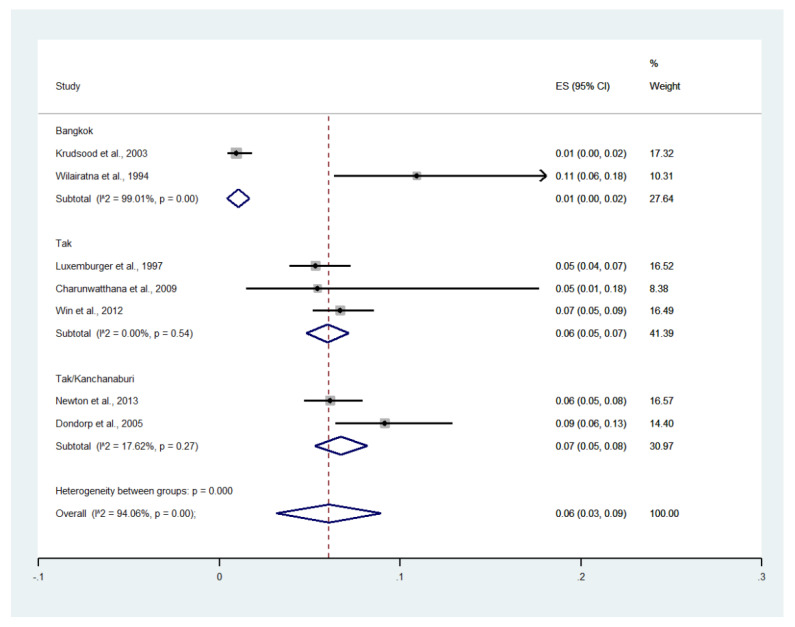
The pooled prevalence of deaths among patients with severe malaria in Thailand. The label for the x-axis is the scale of estimated prevalence. Abbreviations; ES, estimated proportion; CI, confidence interval.

Deaths were reported in eight studies [14,19,21,22,23,24,28,29]. One study conducted at the Hospital for Tropical Diseases (1994) reported no deaths [23]. The remaining seven studies reported 193 deaths among patients with severe malaria. Meta-analysis of the results of seven studies, including a total of 2558 patients, showed that the pooled prevalence of deaths among patients with severe malaria in Thailand was 6% (seven studies; 95% CI: 3–9%, I^2^: 94.06%). Subgroup analysis by province showed that the highest pooled prevalence of mortality among patients with severe malaria was observed in Tak/Kanchanaburi (two studies; 7%, 95% CI: 5–8%, I^2^: 17.62%) and Tak (three studies; 6%, 95% CI: 5–7%, I^2^: 0%). A lower pooled prevalence of mortality was observed in Bangkok (two studies; 1%, 95% CI: 0–2%, I^2^: 99.01%) (Figure 18).

### 3.18. Publication Bias

**Figure 19:**
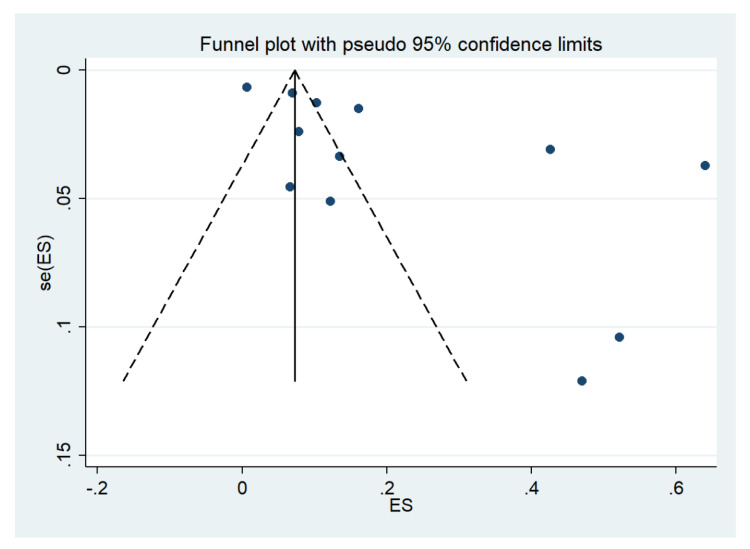
Funnel plot. Abbreviations; es, effect size; se (es), standard error of the es.

**Figure 20:**
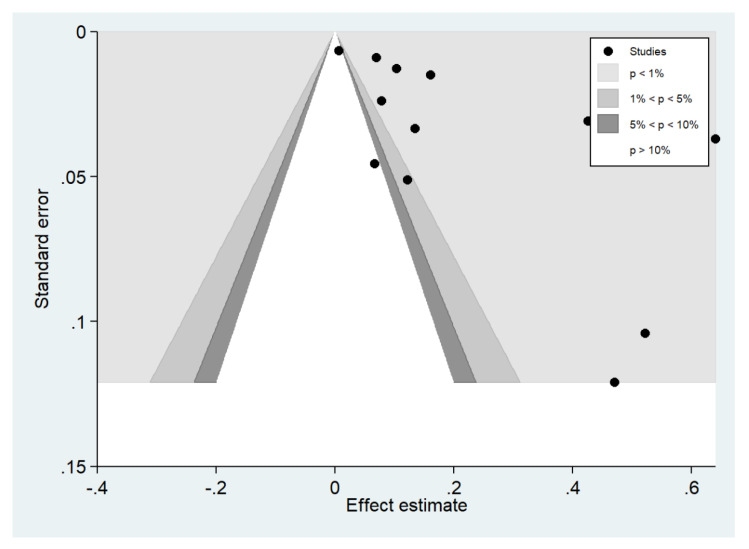
Contour-enhanced funnel plot.

Publication bias was assessed by visualizing funnel plots and using Egger’s test. Funnel plot asymmetry was observed when the effect size and standard error of the effect size for the pooled prevalence of impaired consciousness were plotted using the data from 12 studies (Figure 19). Egger’s test showed significant small-study effects (*p* = 0.014), indicating that the asymmetrical distribution of the funnel plot was related to small-study effects. A contour-enhanced funnel plot indicated that the asymmetrical distribution of the included studies arose from the heterogeneity of the included studies (*p* < 0.01; Figure 20). Publication bias and heterogeneity among the outcomes of included studies might have contributed to the asymmetrical distribution of the funnel plot.

## 4. Discussion

Although *P*. *vivax* (95%) and *P*. *falciparum* (4–5%) were the most common causes of malaria in Thailand in the year 2020 [13], nevertheless, due to Thailand’s National Malaria Elimination Strategy 2017–2026 (NMES), malaria incidence and mortality in Thailand decreased from 2012 to 2020 [30]. The NMES introduced the strategy of robust surveillance and response approach for elimination for each malaria case to deploy needed interventions [31]. Under the strategy, Thai people in several rural communities can access early diagnosis and effective antimalarial treatment. For better-sustained malaria elimination, continuous malaria surveillance and continuous monitoring of patients with malaria are needed to achieve the goal of national malaria control programs and ensure that malaria in Thailand is eliminated, and previous information on clinical status and signs of severity among patients with severe malaria in the past decade is crucial.

The most recent study in Thailand that reported patients with severe malaria was conducted between 2006 and 2012, and those patients were likely to have been referred for treatment to the Hospital for Tropical Diseases in Bangkok [26]. The Hospital for Tropical Diseases was established in 1961 by the late Professor Emeritus Chamlong Harinasuta and the late Professor Khunying Tranakchit Harinasuta and offers services for the treatment of malaria and tropical diseases [32]. Previous studies showed that referrals, no prior history of malaria, body temperature above 38.5 °C, white blood cell count > 10 × 10^9^/mL, presence of schizonts or gametocytemia in peripheral blood smears, and albumin levels of < 3.5 g/dL were associated with increased risks of severe malaria in Thailand [26,33,34]. Furthermore, patients with severe malaria had longer hospital stays and were at increased risk of acquiring other infections than those with uncomplicated malaria [26].

The present study showed that jaundice was the most frequent sign of severity in patients with severe malaria (54%), followed by hyperparasitemia (47%) and impaired consciousness (21%). In South Asian countries, such as India, jaundice (44%) and severe anemia (20%) were reported as predominant signs of severity [35,36]. The results of the present meta-analysis that showed the high proportion of jaundice (54%) was in accordance with the previous study in India, showing most adults with *P*. *falciparum* malaria had jaundice (58.90%) [37]. The difference in the proportions of signs of severity among patients with *P*. *falciparum* malaria in India could be explained by the difference in the age groups of patients. Signs of severity such as jaundice and acute renal failure were common in adults, whereas severe anemia was a predominant sign of severity in children [35]. For severe malaria in South East Asian countries, there are differences in the prevalence of signs of severity. In Singapore, disseminated intravascular coagulation (DIC) (48.5%), renal impairment (36.3%), and hyperparasitemia (36.3%) were commonly found in *P*. *falciparum* malaria, whereas shock (50%) and DIC (37.5%) were commonly found in *P*. *vivax* malaria [38]. In Malaysia, severe malaria was mostly caused by *P*. *knowlesi* (29%), *P*. *vivax* (16%), and *P*. *falciparum* (11%) [39]. In patients with severe *P*. *knowlesi* infections, acute kidney injuries (AKI) (45.6%), jaundice (42%), and hyperparasitaemia (32.5%) were commonly observed [3]. Jaundice (69%), hypotension (46%), and respiratory distress were commonly observed in *P*. *falciparum* malaria, whereas hypotension (71%) and jaundice (29%) were frequently observed in *P*. *vivax* infections [39]. In Cambodia, the most common signs of severity caused *P*. *falciparum* (and a small number of *P*. *vivax*, mixed infection) included prostration (68%), impaired consciousness (65%), and respiratory distress (48%) [40]. In Myanmar, jaundice (46%), coma (20.8%), and acute kidney injury (19%) were the most frequent signs of severity [41]. Meanwhile, cerebral malaria and jaundice were the most frequently severe complications and signs of severity reported in patients with *P*. *falciparum*, respectively [42,43]. Therefore, the most frequent sign of severity observed by the meta-analysis, jaundice (54%), was in accordance with the studies that reported jaundice as the highest prevalence among patients with severe *P*. *falciparum* malaria (69%) in Malaysia [39] and Myanmar [41].

Although *P. vivax* is the most common *Plasmodium* species found in Thailand, patients with severe *P. vivax* in Thailand were rarely reported [44]. Therefore, data on signs of severity among patients with *P. vivax* in Thailand are not available for inclusion in the present meta-analysis. In Africa, where malaria is endemic, such as Ethiopia, severe anemia (42%) and hyperpyrexia (21%) were the most common signs of severity in patients with *P. vivax* malaria [45]. Another study conducted in the same areas demonstrated that prostration (31%), hyperpyrexia (24%), and severe anemia (13.8%) were predominant signs of severity [46]. In Asian countries, such as India, hepatic dysfunction (29%), renal dysfunction (21%), and cerebral malaria (16.1%) were common in patients with *P. vivax* malaria [47]. The meta-analysis of 49 studies that reported severe *P. vivax* malaria demonstrated that severe anemia, jaundice, and respiratory distress were frequently observed [8].

The present study had several limitations. First, the prevalence and proportions of each sign of severity were heterogeneous among the included studies. Therefore, random-effect models were applied for all meta-analyses. With the limitation of the high heterogeneity between studies, careful interpretations of the meta-analysis results were recommended. Second, the period under analysis ends in 2012 due to the limited number of studies reporting signs of the severity of malaria in Thailand because the prevalence of malaria in Thailand has continuously decreased in recent decades. In addition, no severe malaria in Thailand has been reported since 2014 [48]. Third, data from a single hospital in Bangkok is not representative of the entire country. In addition, other hospitals in other regions of Thailand did not report severe malaria in the literature. Therefore, those patients could not be included in the systematic reviews. Additionally, information on severe malaria in Thailand was limited. Fourth, there were limitations in the definitions of signs of severity, such as impaired consciousness, which was later defined by the WHO criteria for severe malaria [5]. Therefore, studies conducted before the introduction of the WHO criteria for severe malaria [14,20,23,27,29] did not report the impaired consciousness but reported “cerebral malaria”, which might cover one or more signs of severity, such as prostration, impaired consciousness, and convulsion. These differences in definitions of signs of severity might affect the pooled prevalence of the signs of severity among patients with severe malaria in Thailand.

## 5. Conclusions

The present study revealed that jaundice, hyperparasitemia, and impaired consciousness/coma were the most common signs of severity identified in the Thai population with malaria. Data from the meta-analysis results are essential in managing patients with severe malaria in Thailand, with particular attention to the management of signs of severity, such as monitoring and managing jaundice, monitoring patients’ parasite status, or giving parenteral antimalarial treatment for patients with hyperparasitemia, and close observation of unconscious patients. The effective management of patients with malaria will reduce the signs of severity and prevent mortality which promotes malaria-elimination efforts in Thailand.

## Figures and Tables

**Table 1:** Characteristics of the included studies.

Author	Year Conducted	Study Sites	Province	Study Design	N Participants	Age Group	% Male	Died	Prostration	Impaired Con/Coma	Convulsion	Severe Anemia	Jaundice	Renal Impairment	Shock/Hypotension	Hyperparasitemia	Acidosis	Pulmonary edema/ARDS	DIC/Bleeding	Hypoglycemia	Total Signs of Severity	%Cutoff Parasitemia
Charunwatthana et al., 2009	2003–2005	Mae Sot Hospital	Tak	RCT	23	Adults patients	NS	2	NS	12	NS	2	6	2	1	7	3	4	NS	NS	37	>10%
Chuncharunee et al., 1997	1992–1994	Ramathibodi Hospital, Pramongkutklao Hospital	Bangkok	Prospective observational study	17	Adults patients	100	NS	NS	8	NS	NS	NS	10	NS	6	NS	6	1	NS	31	>10%
Dondorp et al., 2005	NS	Sangklaburi Hospital, Mae Sot Hospital	Tak/ Kanchanaburi	Prospective observational study	167	Adults patients	NS	28	NS	107	NS	10	87	22	8	72	NS	NS	NS	NS	306	>10%
Krudsood et al., 2003	1993–1996	Hospital for Tropical Diseases	Bangkok	Clinical trials	803	Adults patients	NS	8	NS	56	NS	116	340	26	NS	332	NS	NS	NS	NS	870	>2%
Looareesuwan et al., 1995	1994	Hospital for Tropical Diseases	Bangkok	Clinical trials	30	Adults patients	76.7	NS	NS	2	NS	NS	6	1	NS	24	NS	NS	NS	NS	33	>4%
Luxemburger et al., 1997	1994	Shoklo hospital	Tak	Prospective observational study	609	Children and adults	56	37	NS	98	89	98	NS	NS	NS	409	NS	NS	NS	NS	694	>4%
Newton et al., 2013	1986–2002	Hospitals in Kanchanaburi (1986–1993), Hospitals in Sangklaburi (1994–1995), Hospitals in Mae Sot (1995–2002)	Tak/ Kanchanaburi	Retrospective observational study	571	Adults patients		51	NS	59	2	12	341	57	2	283	63	8	3	5	835	>4%
Niphakasem B, 2006	2003–2006	Somdejt Prachaotaksin Maharaj Hospital	Bangkok	Retrospective observational study	41	Children		NS	24	5	1	3	NS	2	2	5	3	2	NS	NS	23	>4%
Sagaki et al., 2013	2006–2012	Hospital for Tropical Diseases	Bangkok	Retrospective case–control study	104	Adults patients		NS	NS	14	NS	3	67	20	27	44	17	21	6	2	221	>2%
Wilairatna et al., 1994	1991	Hospital for Tropical Diseases	Bangkok	Retrospective observational study	127	Children and adults		12	NS	10	NS	NS	124	12	3	83	NS	2	NS	NS	110	>4%
Wilairatna et al., 2000	1999	Hospital for Tropical Diseases	Bangkok	Clinical trials	150	Children and adults	66.7	NS	NS	1	NS	13	103	3	NS	70	NS	NS	NS	NS	190	>2%
Win et al., 2012	2004–2008	Mae Sot Hospital	Tak	Retrospective observational study	258	Adults patients	73.6	55	178	110	30	16	139	115	86	NS	109	23	7	13	826	NS

Abbreviation: NS, not specified.

**Table 2:** The pooled prevalence of each sign of severity among patients with severe malaria in Thailand (total number of patients with severe malaria = 2900).

Signs of Severity *	Estimated Prevalence (%) **	95% CI (%)	I^2^	Number of Patients in Each Sign of Severity	Number of Patients with Severe Malaria	Number of Studies for Estimation
Jaundice	54	36–72	99.02	1213	2233	9
Hyperparasitemia	47	38–56	95.07	1335	2642	11
>2%	42	39–45	0	446	1057	3
>4%	55	39–71	96.96	804	1378	5
>10%	41	34–48	0	85	207	3
Impaired consciousness/coma	21	14–28	97.96	482	2900	12
Acidosis	18	5–31	95.62	195	997	5
Renal impairment	13	8–19	95.81	270	2291	11
Shock	10	4–17	96.47	129	1291	7
Convulsions	9	2–19	98.41	121	1479	3
Severe anemia	8	4–12	94.26	273	2726	9
Pulmonary edema/ARDS	8	4–12	88.15	66	1141	7
Hypoglycemia	4	1–8	89.02	20	933	3
Bleeding/DIC	2	1–5	74.38	17	950	4

* Signs of severity by guidelines for the treatment of malaria 2015, and WHO Guidelines for malaria 2021, except for hyperparasitemia >2% and >4% adapted guidelines. ** The pooled prevalence was estimated by the random-effects model.

**Table 3:** The estimated proportion of signs of severity per all signs of severity in Thailand (total signs of severity = 4324).

Signs of Severity *	Estimated Proportion (%) **	95% CI (%)	I^2^	Number of Each Sign of Severity	Total Number of Signs of Severity for Estimation	Number of Studies for Estimation
Hyperparasitemia	33	25–42	97.29	1335	3498	11
>2%	32	20–44	94.20	446	1281	3
>4%	42	26–58	97.72	804	1843	5
>10%	23	18–27	0	85	374	3
Jaundice	33	24–43	97.07	1213	3552	9
Impaired consciousness/coma	12	8–16	96.55	482	4324	12
Acidosis	9	6–12	75.27	195	1966	5
Renal impairment	7	4–9	90.32	270	3630	11
Severe anemia	6	3–9	95.57	273	4026	9
Convulsions	5	0–11	98.32	121	2355	3
Shock	5	2–8	95.21	129	2506	7
Pulmonary edema/ARDS	3	1–5	83.21	66	1141	7
Bleeding/DIC	1	0–2	52.70	17	1913	4
Hypoglycemia	1	0–2	45.60	20	1882	3

* Signs of severity by guidelines for the treatment of malaria 2015, and WHO Guidelines for malaria 2021, except for hyperparasitemia >2% and >4% (adapted guidelines). ** The proportion was estimated by the random-effects model.

## Data Availability

All data in this study were provided in the main manuscript and supplementary files.

## Supplementary Materials

The following are available online at https://www.mdpi.com/article/10.3390/ijerph19031196/s1, Supplementary Figure S1, The estimated proportion of jaundice per all signs of severity; Supplementary Figure S2, The subgroup analysis of the estimated proportion of jaundice per all signs of severity.; Supplementary Figure S3, The estimated proportion of the hyperparasitemia per all signs of severity at different cutoffs; Supplementary Figure S4, The estimated proportion of impaired consciousness per all signs of severity; Supplementary Figure S5, The subgroup analysis of the estimated proportion of impaired consciousness per all signs of severity; Supplementary Figure S6, The estimated proportion of acidosis per all signs of severity; Supplementary Figure S7, The estimated proportion of renal impairment per all signs of severity; Supplementary Figure S8, Subgroup analysis of the estimated proportion of renal impairment per all signs of severity; Supplementary Figure S9, The estimated proportion of severe anemia per all signs of severity; Supplementary Figure S10, Subgroup analysis of the estimated proportion of severe anemia per all signs of severity; Supplementary Figure S11, The estimated proportion of convulsion per all signs of severity; Supplementary Figure S12, The estimated proportion of shock per all signs of severity; Supplementary Figure S13, The estimated proportion of pulmonary edema/ARDS per all signs of severity; Supplementary Figure S14, The estimated proportion of bleeding/DIC per all signs of severity; Supplementary Figure S15, The estimated proportion of hypoglycemia per all signs of severity; Table S1, search terms; Table S2, quality of the included studies.
