# Supplementary material for: Prevalence of Signs of Severity Identified in the Thai Population with Malaria: A Systematic Review and Meta-Analysis
Source: Int J Environ Res Public Health. 2022 Jan 21;19(3):1196. doi: 10.3390/ijerph19031196 (PMC8834971; doi:10.3390/ijerph19031196)
Supplement: Supplementary file 1 [file ijerph-19-01196-s001.zip › Table S1. Search term.pdf]

Prevalence of signs of severity identified in the Thai population with malaria: a systematic review and meta-analysis

Wanida Mala [1], Polrat Wilairatana [2], Chutharat Samerjai [1], Frederick Ramirez Masangkay [3], Kwuntida Uthaisar Kotepui [1] and Manas Kotepui [1,*]

[1]Medical Technology, School of Allied Health Sciences, Walailak University, Tha Sala, Nakhon Si Thammarat, Thailand; kwuntida.ut@wu.ac.th

[2]Department of Clinical Tropical Medicine, Faculty of Tropical Medicine, Mahidol University, Bangkok, Thailand; polrat.wil@mahidol.ac.th

[3]Department of Medical Technology, Faculty of Pharmacy, University of Santo Tomas, Manila, Philippines; frederick_masangkay2002@yahoo.com

*Correspondence: manas.ko@wu.ac.th; Tel.: +66954392469

Table S1. Search term

**Databases**	**Search terms/Search strategy**	**Date**
MEDLINE	("severe malaria" OR "complicated malaria") AND (Thai OR Thailand OR Siam)<br><br>Search results: 299	8 August 2021

Scopus	("severe malaria" OR "complicated malaria") AND (Thai OR Thailand OR Siam)<br><br>Search option: Title, abstract, keywords<br><br>Search results: 142	8 August 2021
Web of Science	("severe malaria" OR "complicated malaria") AND (Thai OR Thailand OR Siam)<br><br>Search option: All fields<br><br>Search results: 300	8 August 2021
