# Supplementary material for: Prevalence of Signs of Severity Identified in the Thai Population with Malaria: A Systematic Review and Meta-Analysis
Source: Int J Environ Res Public Health. 2022 Jan 21;19(3):1196. doi: 10.3390/ijerph19031196 (PMC8834971; doi:10.3390/ijerph19031196)
Supplement: Supplementary file 1 [file ijerph-19-01196-s001.zip › Table S2. Quality of the included studies.pdf]

Prevalence of signs of severity identified in the Thai population with malaria: a systematic review and meta-analysis

Wanida Mala [1], Polrat Wilairatana [2], Chutharat Samerjai [1], Frederick Ramirez Masangkay [3], Kwuntida Uthaisar Kotepui [1] and Manas Kotepui [1,*]

[1]Medical Technology, School of Allied Health Sciences, Walailak University, Tha Sala, Nakhon Si Thammarat, Thailand; kwuntida.ut@wu.ac.th

[2]Department of Clinical Tropical Medicine, Faculty of Tropical Medicine, Mahidol University, Bangkok, Thailand; polrat.wil@mahidol.ac.th

[3]Department of Medical Technology, Faculty of Pharmacy, University of Santo Tomas, Manila, Philippines; frederick_masangkay2002@yahoo.com

*Correspondence: manas.ko@wu.ac.th; Tel.: +66954392469

Table S2. Quality of the included studies

JBI Critical Appraisal Checklist for Randomized Controlled Trials

	Study	Score (out of 13)	Score (percentage)	Quality
1.	Charunwatthana et al., 2009	13	100	High
2.	Krudsood et al., 2003	7	53	Moderate or low-quality study

3.	Looareesuwan et al., 1995	7	53	Moderate or low-quality study
4.	Wilairatna et al., 2000	7	53	Moderate or low-quality study

JBI Critical Appraisal Checklist for Studies Reporting Prevalence Data

	**Study**	**Score (out of 9)**	**Score (percentage)**	**Quality**
1.	Chuncharunee et al. 1997	7	78	High
2.	Dondorp et al., 2005	9	100	High
3.	Luxemburger et al., 1997	9	100	High
4.	Newton et al., 2013	9	100	High
5.	Niphakasem B., 2006	7	78	High
6.	Sagaki et al., 2013	9	100	High
7.	Wilairatana et al., 1994	8	89	High
8.	Win et al., 2012	9	100	High
