# Supplementary figures and images for: Prevalence of Signs of Severity Identified in the Thai Population with Malaria: A Systematic Review and Meta-Analysis
Source: Int J Environ Res Public Health. 2022 Jan 21;19(3):1196. doi: 10.3390/ijerph19031196 (PMC8834971; doi:10.3390/ijerph19031196)

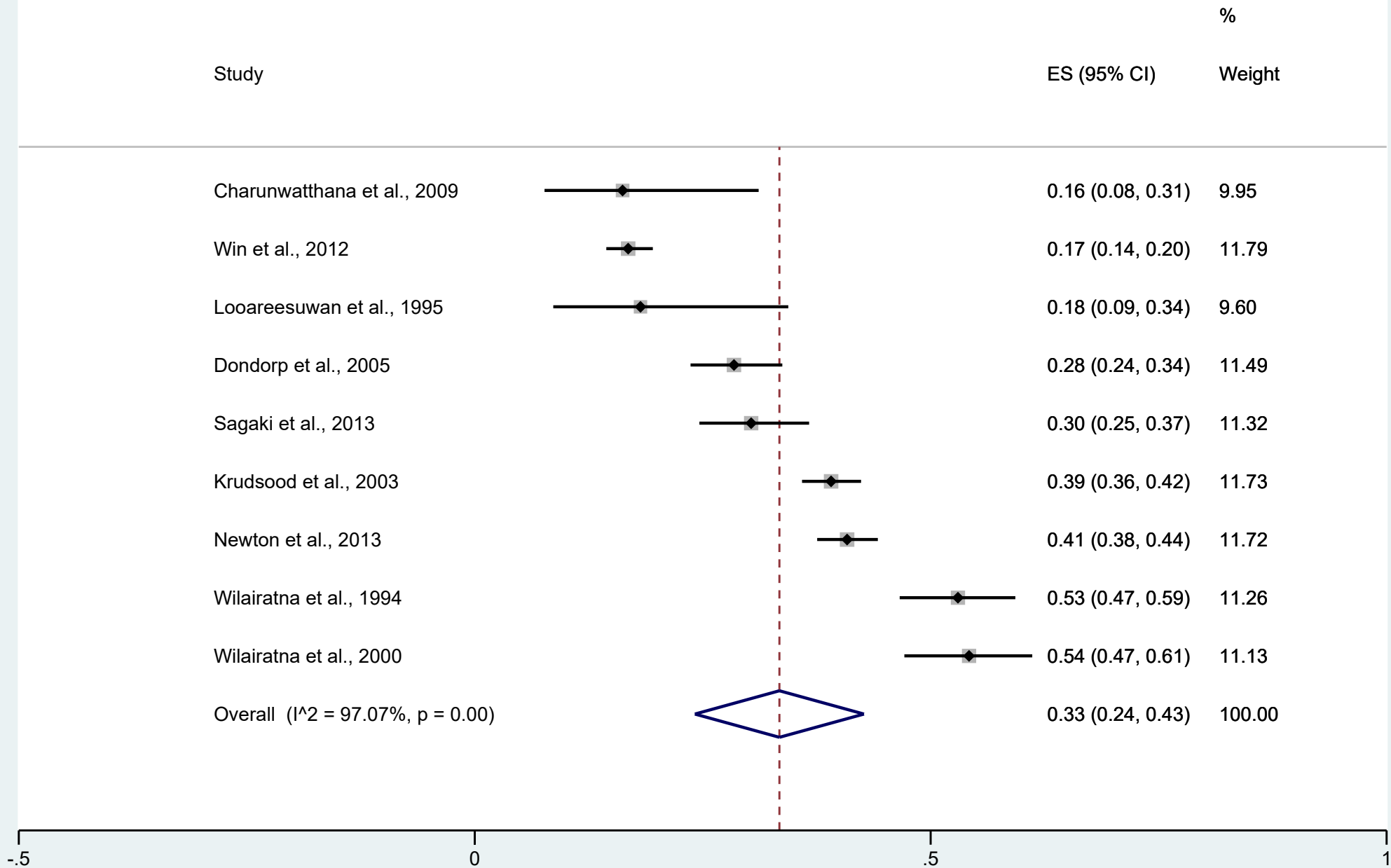

Supplement: Supplementary file 1 [file ijerph-19-01196-s001.zip › Supplementary Fig 1. Jaundice no subgroup.pdf]

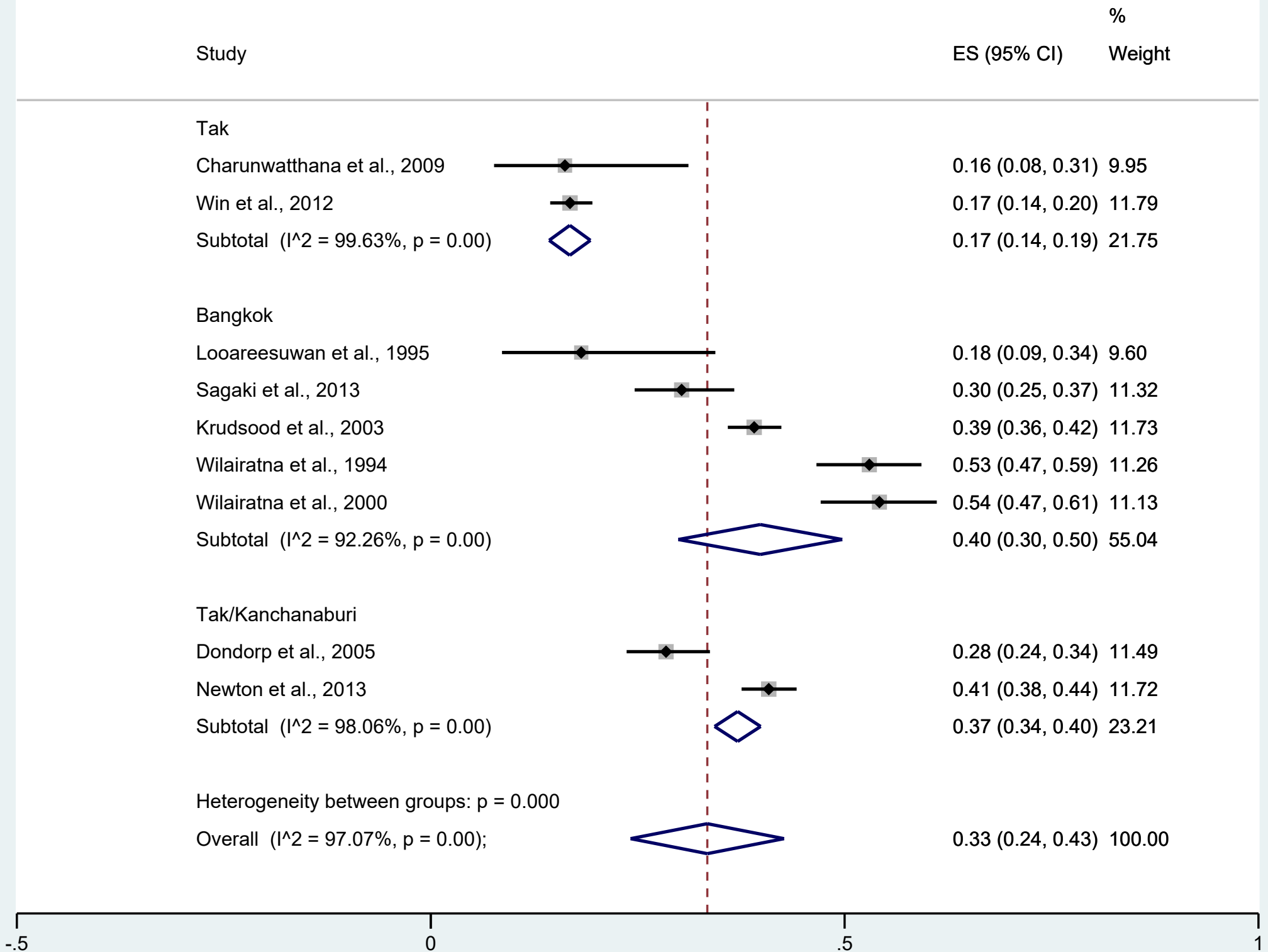

Supplement: Supplementary file 1 [file ijerph-19-01196-s001.zip › Supplementary Fig 2. Jaundice with subgroup.pdf]

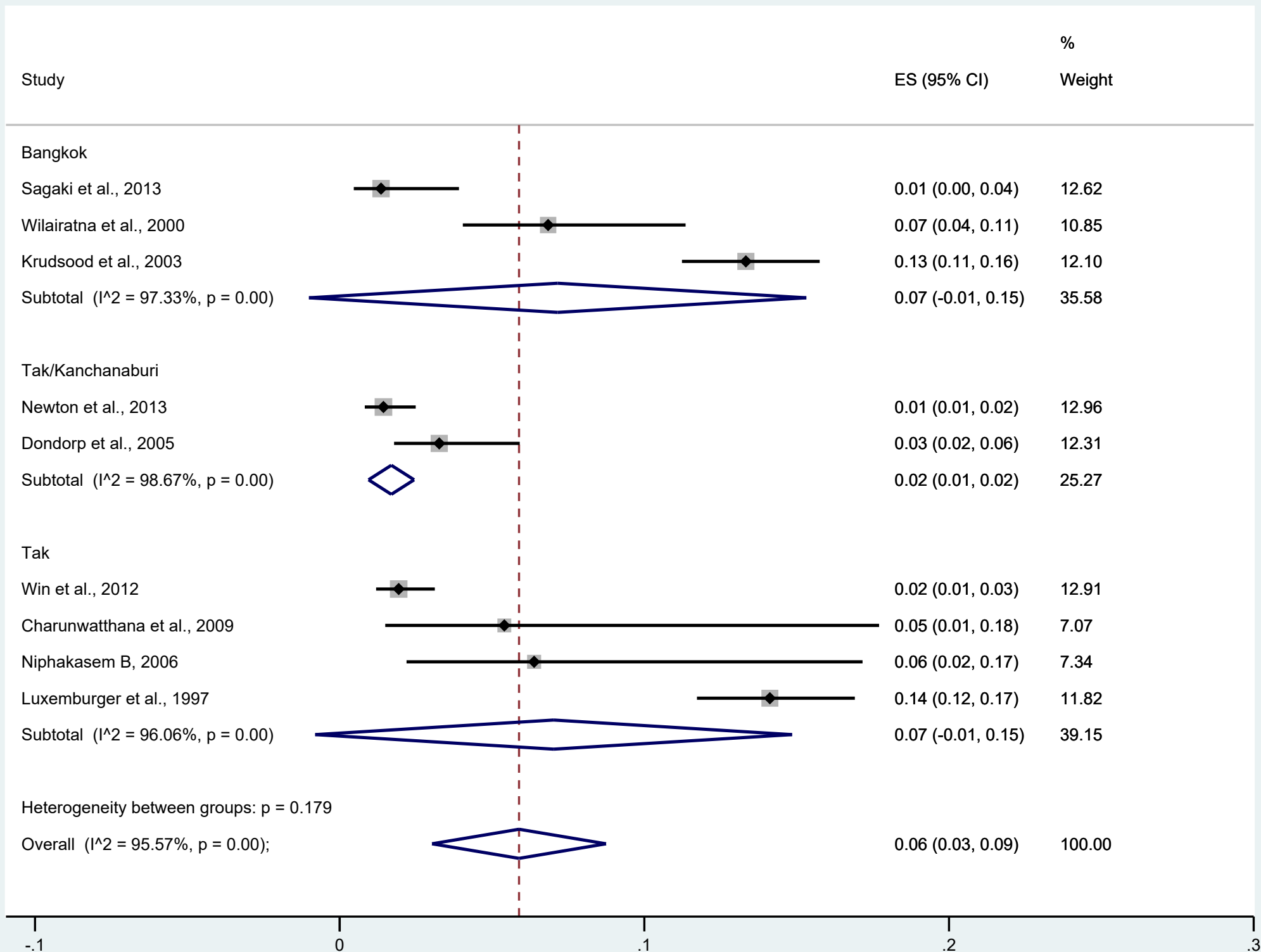

Supplement: Supplementary file 1 [file ijerph-19-01196-s001.zip › Supplementary Fig. 10. Severe anemia with subgroup.pdf]

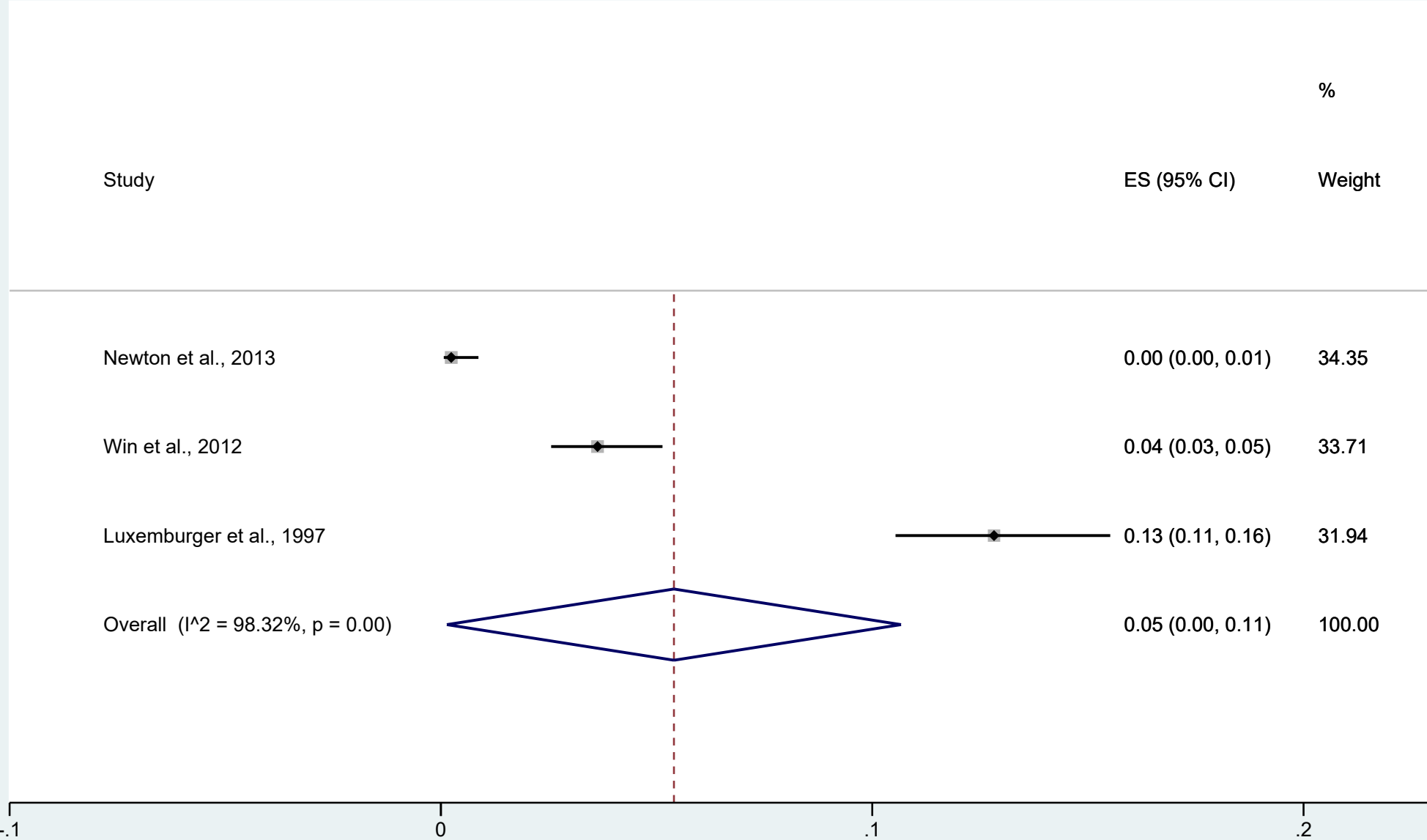

Supplement: Supplementary file 1 [file ijerph-19-01196-s001.zip › Supplementary Fig. 11. Convulsion.pdf]

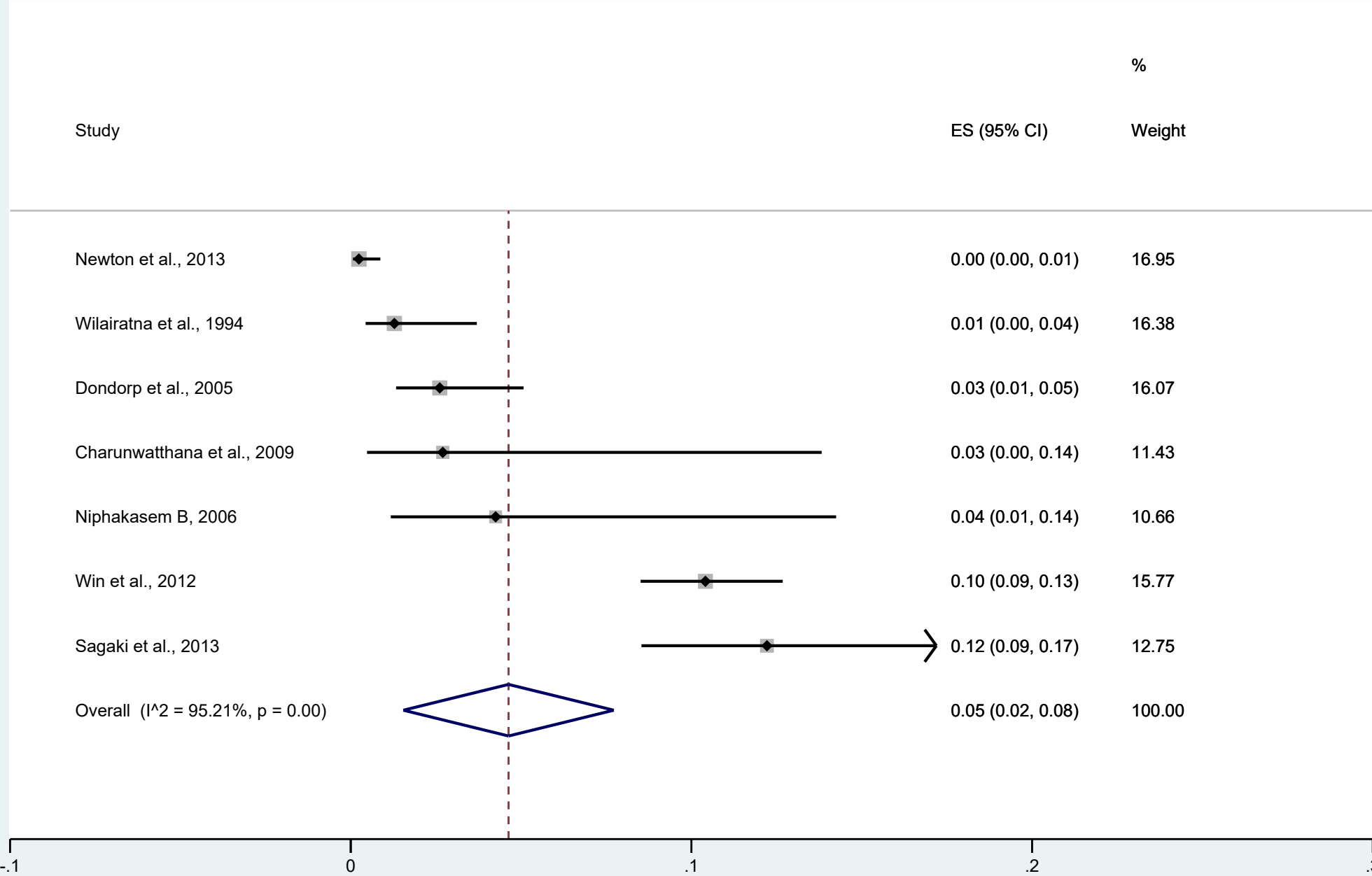

Supplement: Supplementary file 1 [file ijerph-19-01196-s001.zip › Supplementary FIg. 12. Shock.pdf]

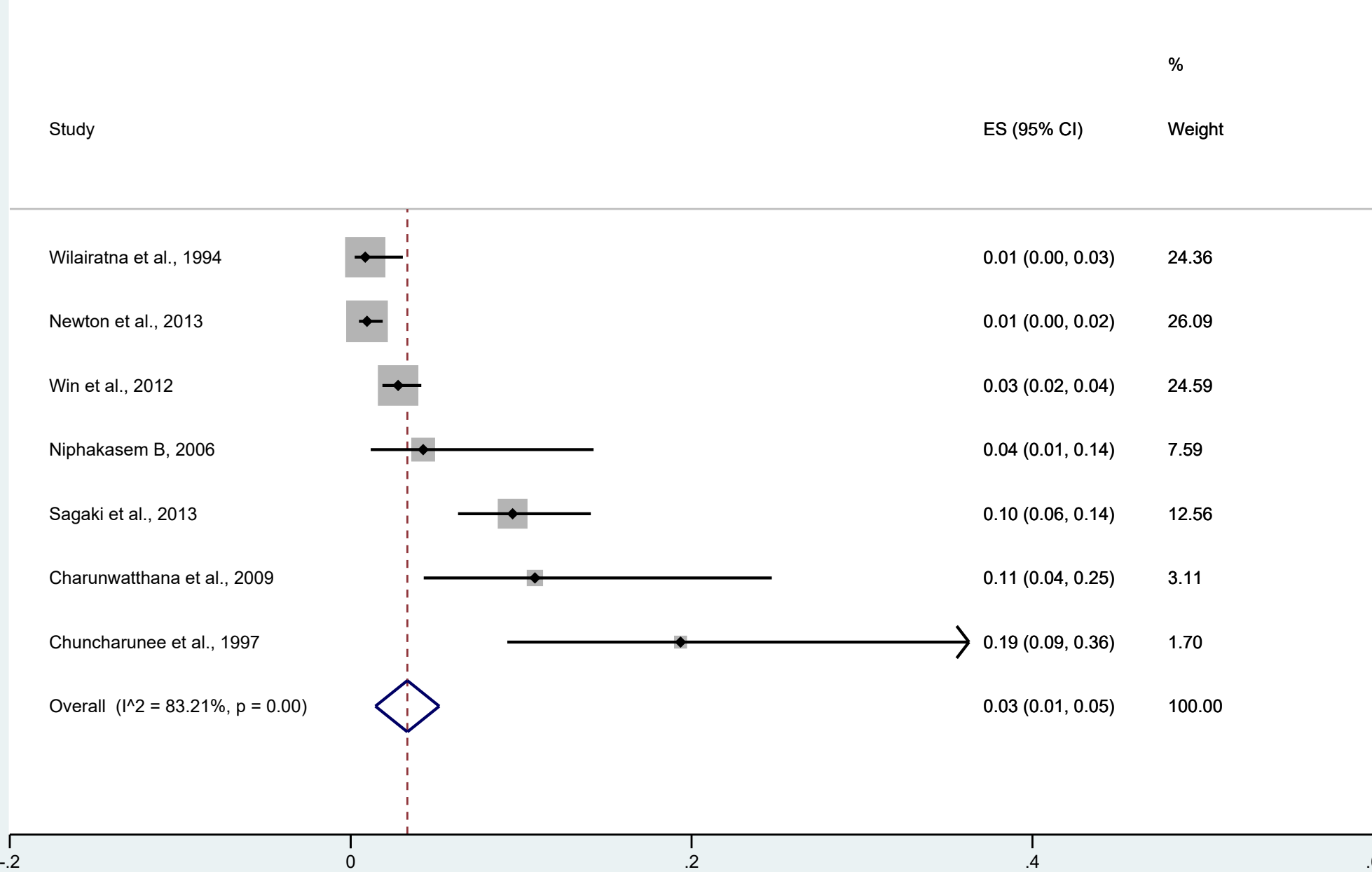

Supplement: Supplementary file 1 [file ijerph-19-01196-s001.zip › Supplementary Fig. 13. Pulmonary edema.pdf]

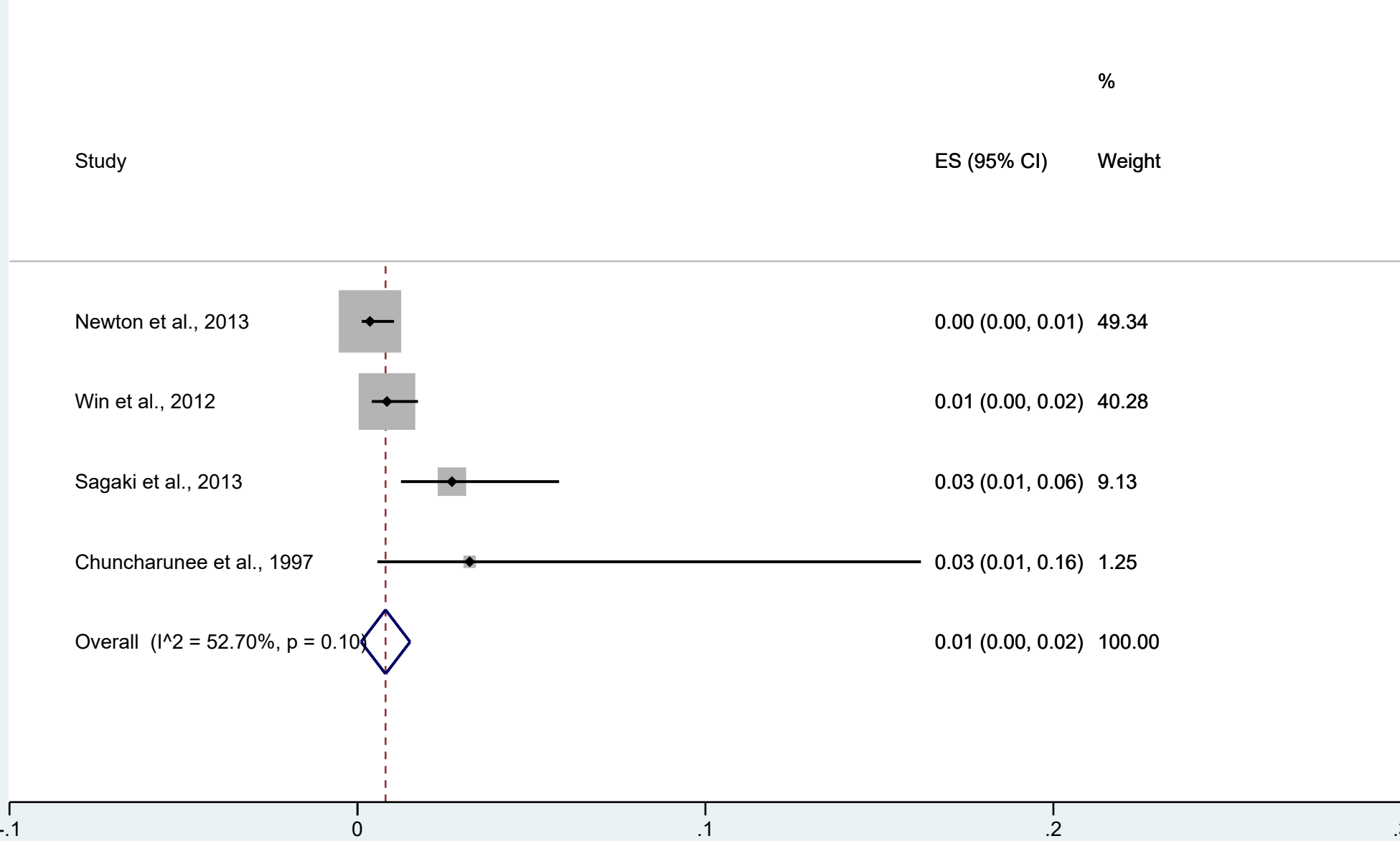

Supplement: Supplementary file 1 [file ijerph-19-01196-s001.zip › Supplementary Fig. 14 Bleeding.pdf]

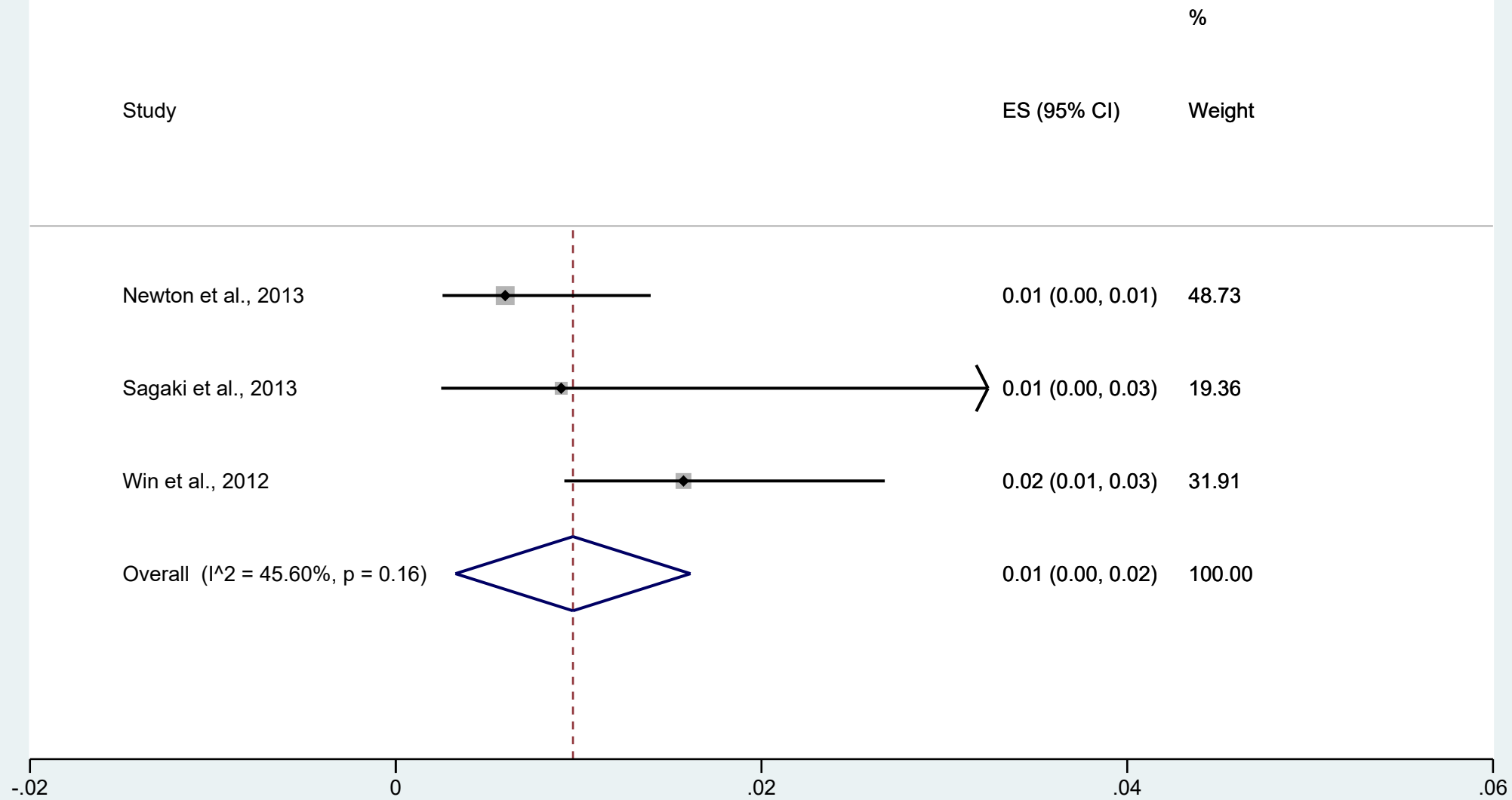

Supplement: Supplementary file 1 [file ijerph-19-01196-s001.zip › Supplementary Fig. 15. Hypoglycemia.pdf]

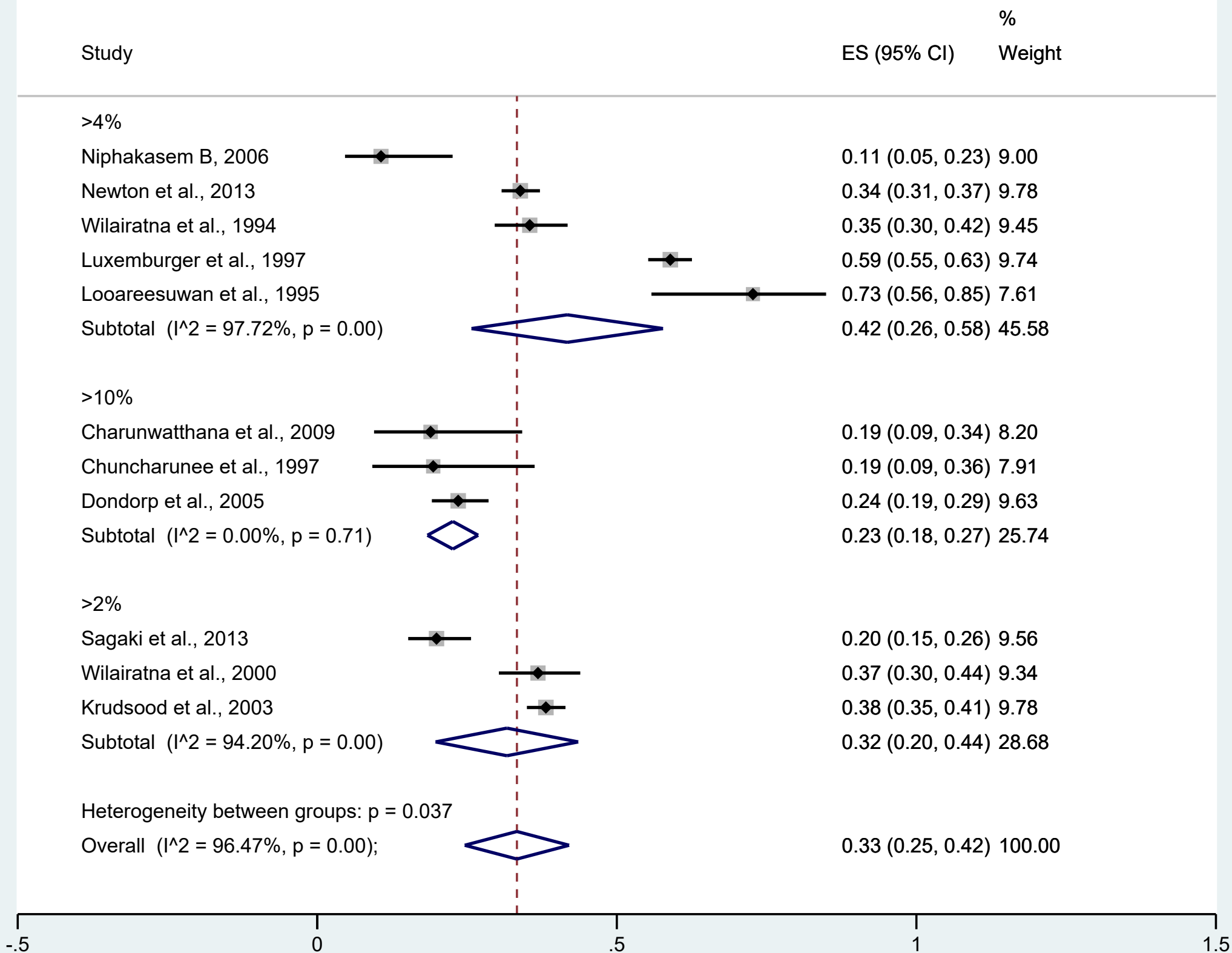

Supplement: Supplementary file 1 [file ijerph-19-01196-s001.zip › Supplementary Fig. 3. Hyperparasitemia.pdf]

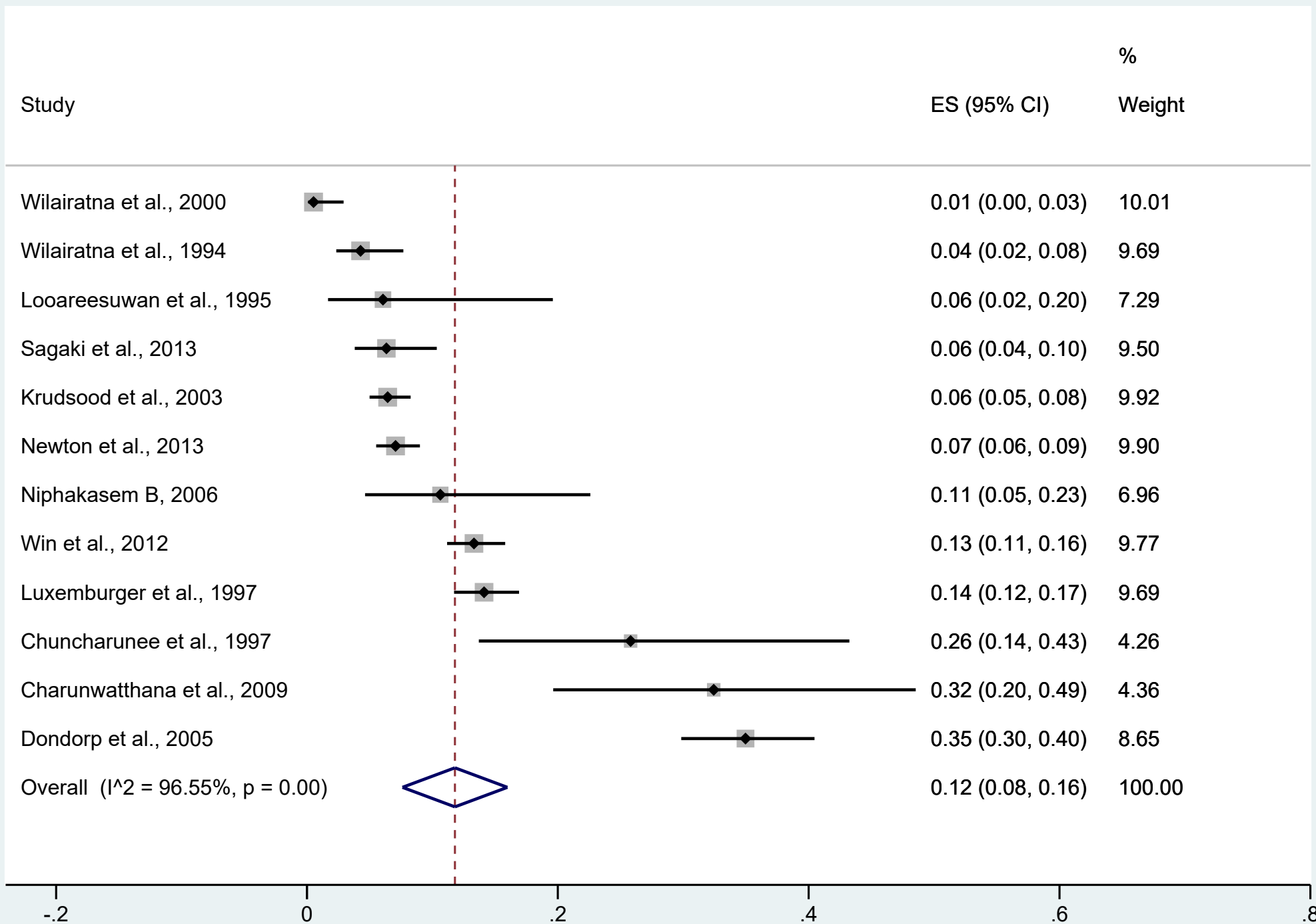

Supplement: Supplementary file 1 [file ijerph-19-01196-s001.zip › Supplementary Fig. 4. Impaired conciousness no subgroup.pdf]

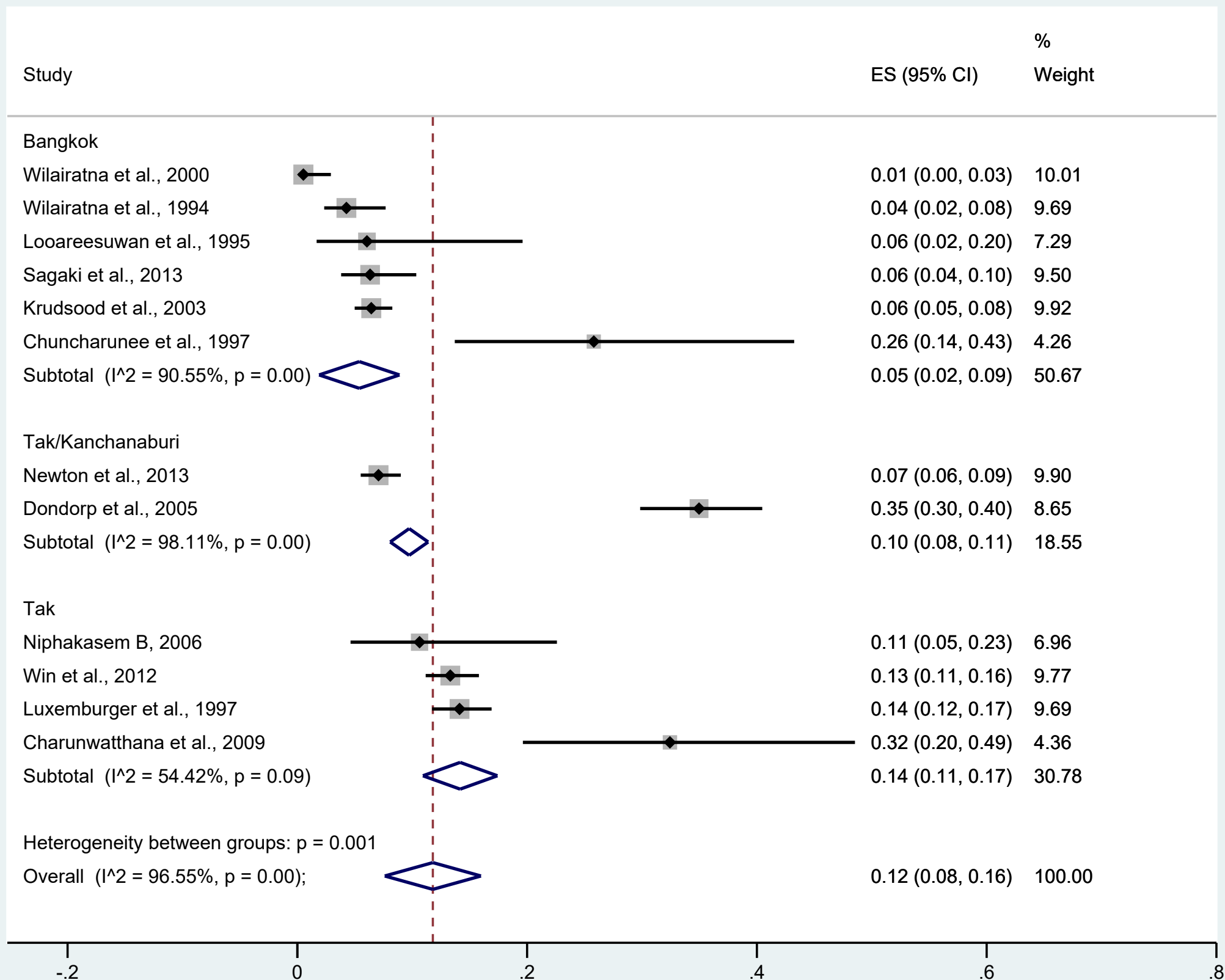

Supplement: Supplementary file 1 [file ijerph-19-01196-s001.zip › Supplementary Fig. 5. Impaired conciousness with subgroup.pdf]

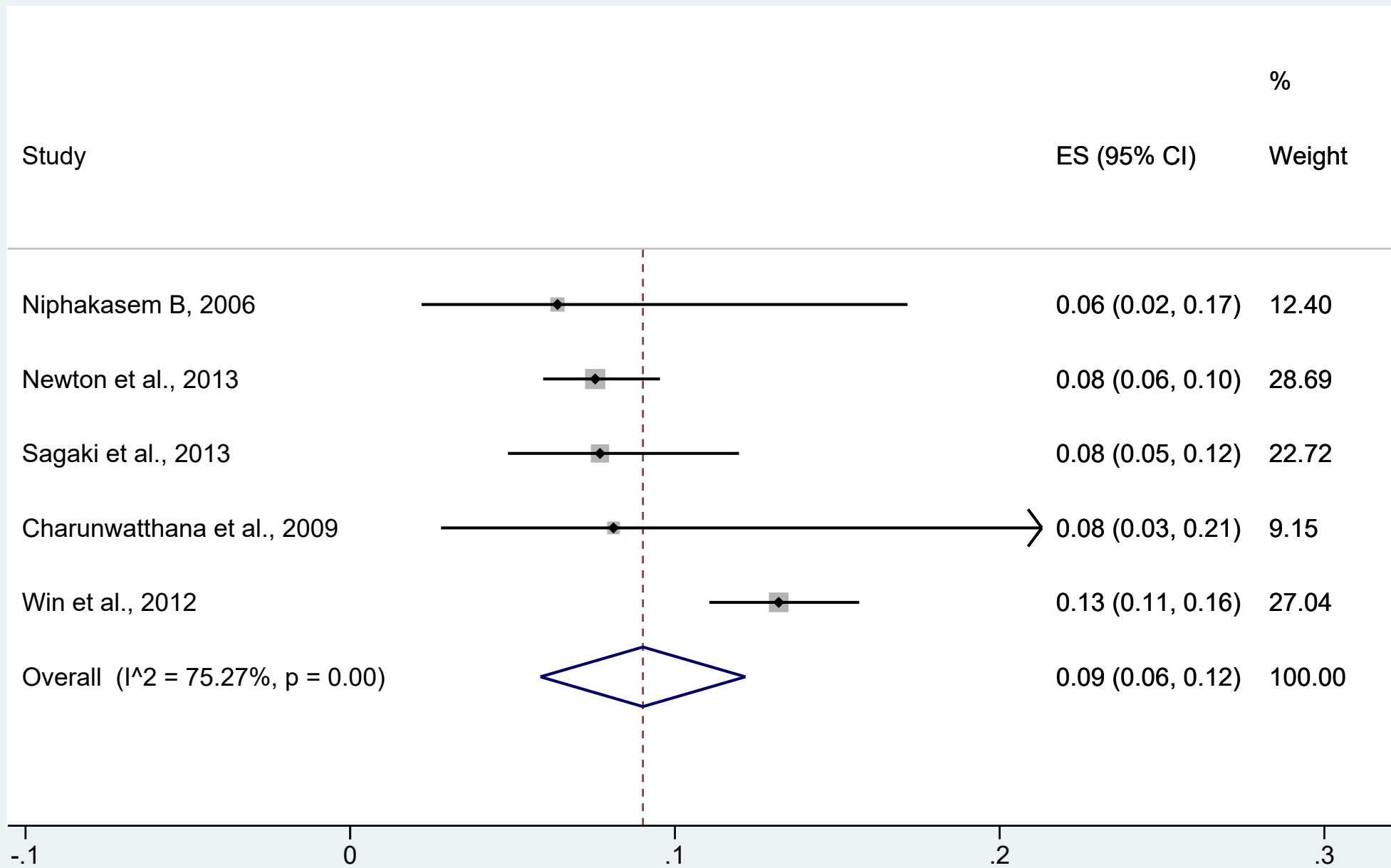

Supplement: Supplementary file 1 [file ijerph-19-01196-s001.zip › Supplementary Fig. 6. Acidosis.pdf]

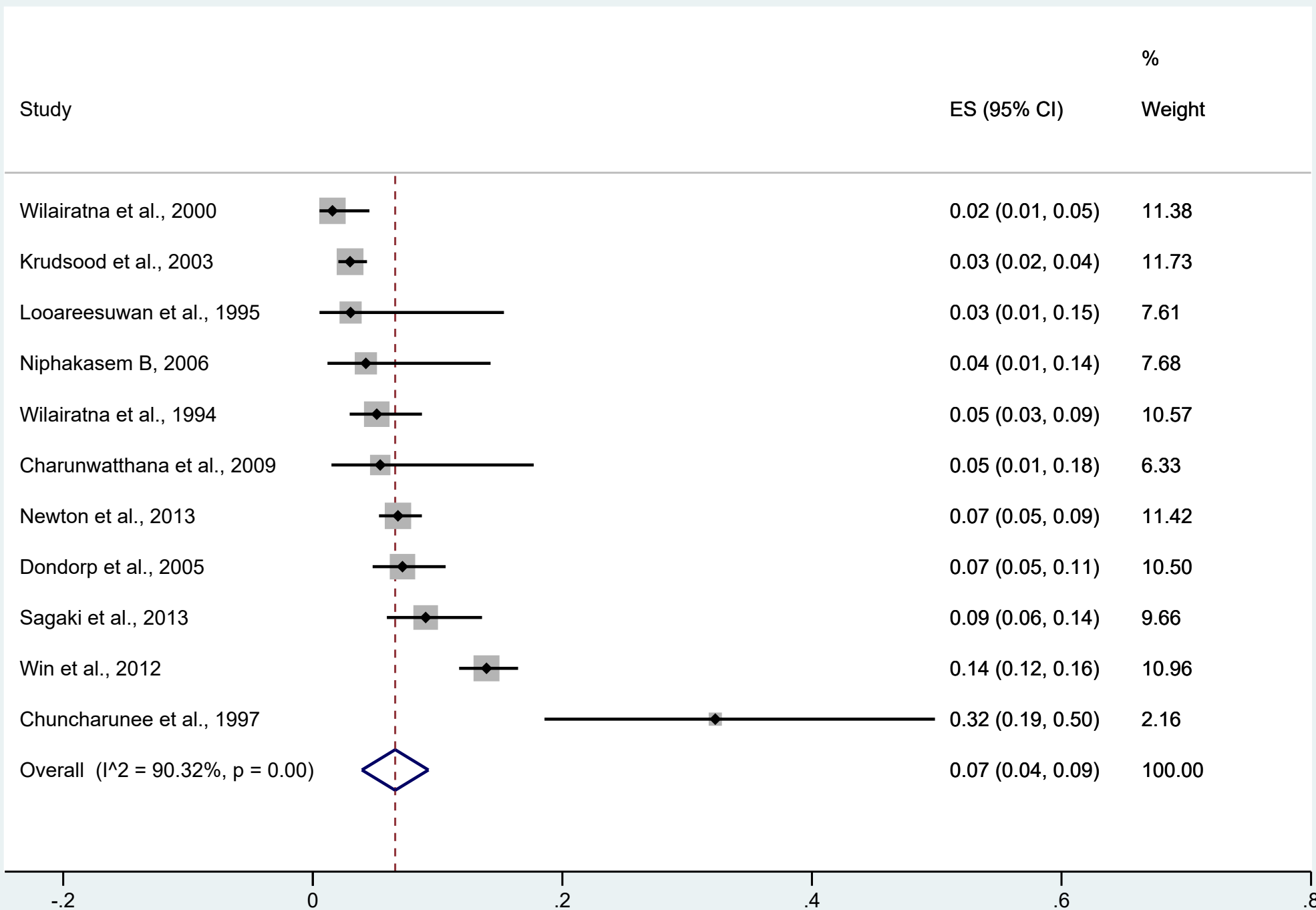

Supplement: Supplementary file 1 [file ijerph-19-01196-s001.zip › Supplementary Fig. 7. Renal impairment no subgroup.pdf]

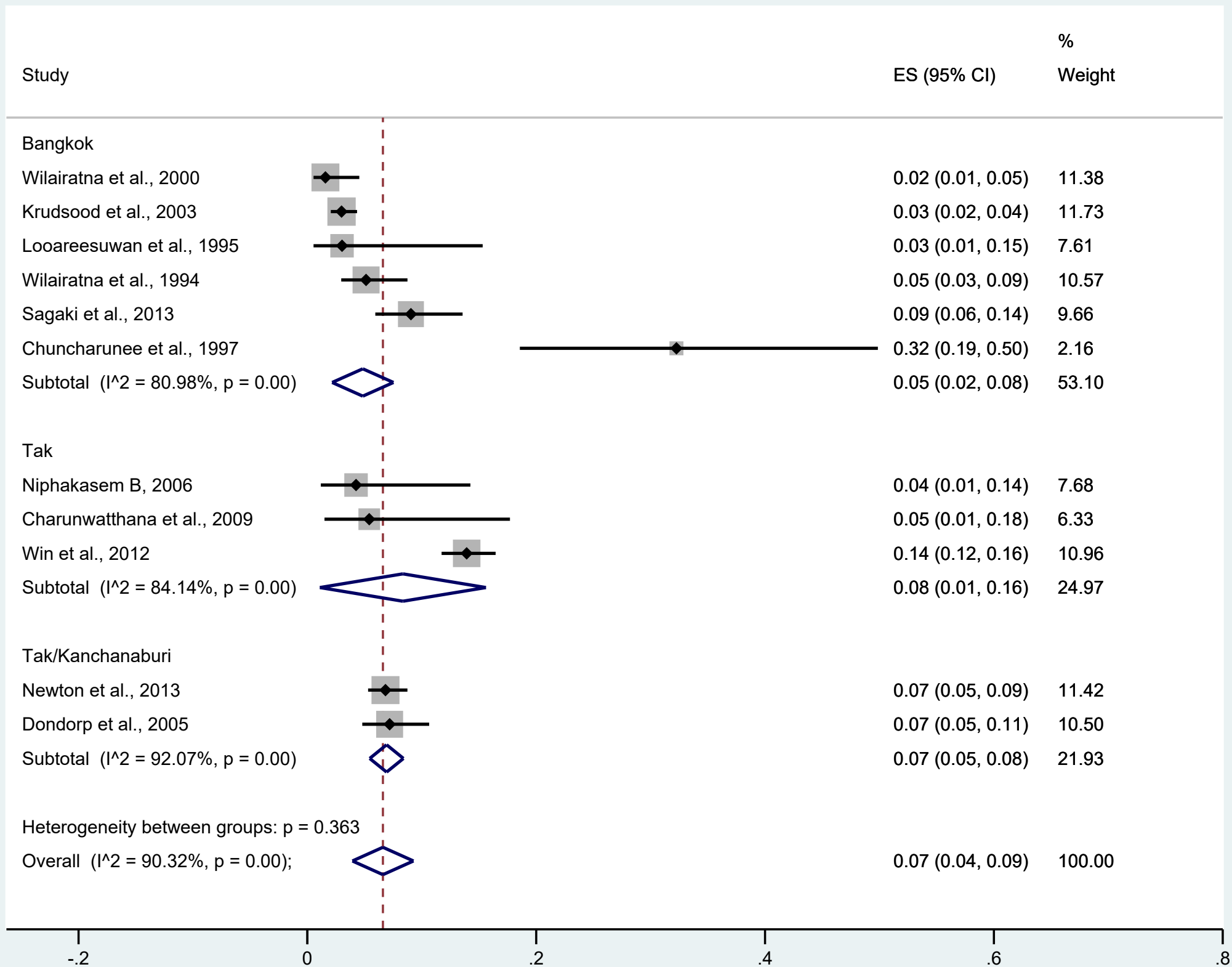

Supplement: Supplementary file 1 [file ijerph-19-01196-s001.zip › Supplementary Fig. 8. Renal impairment with subgroup.pdf]

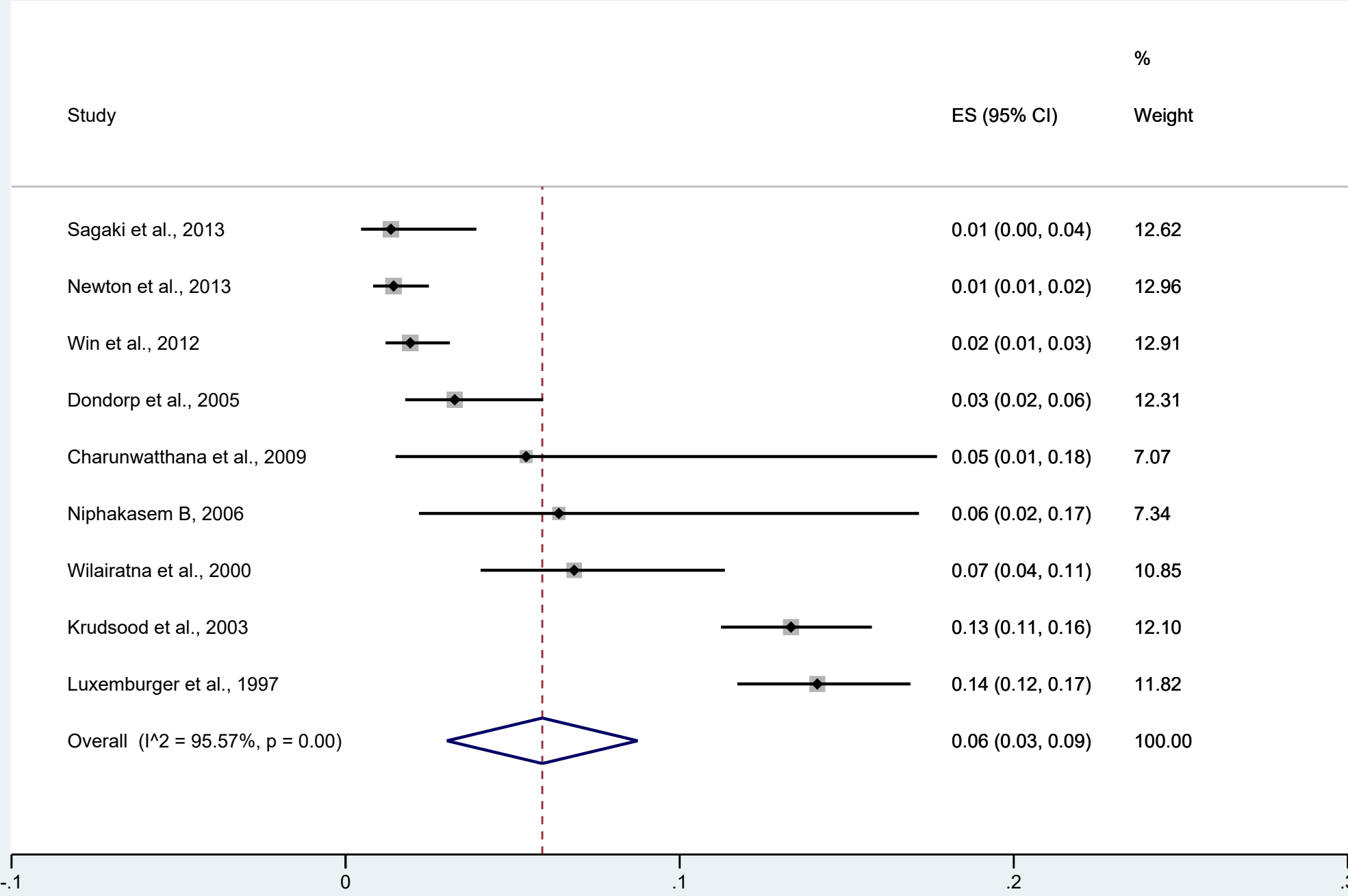

Supplement: Supplementary file 1 [file ijerph-19-01196-s001.zip › Supplementary Fig. 9. Severe anemia no subgroup.pdf]
